# Predicting conversion from mild cognitive impairment to Alzheimer’s disease: a multimodal approach

**DOI:** 10.1093/braincomms/fcae208

**Published:** 2024-06-14

**Authors:** Daniel Agostinho, Marco Simões, Miguel Castelo-Branco

**Affiliations:** Coimbra Institute for Biomedical Imaging and Translational Research (CIBIT), ICNAS, Faculty of Medicine, University of Coimbra, 3000-548 Coimbra, Portugal; Faculty of Science and Technology, Centre for Informatics and Systems of the University of Coimbra (CISUC), 3030-790 Coimbra, Portugal; Intelligent Systems Associate Laboratory (LASI), 4800-058 Guimarães, Portugal; Coimbra Institute for Biomedical Imaging and Translational Research (CIBIT), ICNAS, Faculty of Medicine, University of Coimbra, 3000-548 Coimbra, Portugal; Faculty of Science and Technology, Centre for Informatics and Systems of the University of Coimbra (CISUC), 3030-790 Coimbra, Portugal; Intelligent Systems Associate Laboratory (LASI), 4800-058 Guimarães, Portugal; Coimbra Institute for Biomedical Imaging and Translational Research (CIBIT), ICNAS, Faculty of Medicine, University of Coimbra, 3000-548 Coimbra, Portugal; Intelligent Systems Associate Laboratory (LASI), 4800-058 Guimarães, Portugal

**Keywords:** Alzheimer’s disease, multi-modal classification, sensitive biomarkers, ensemble learning

## Abstract

Successively predicting whether mild cognitive impairment patients will progress to Alzheimer’s disease is of significant clinical relevance. This ability may provide information that can be leveraged by emerging intervention approaches and thus mitigate some of the negative effects of the disease. Neuroimaging biomarkers have gained some attention in recent years and may be useful in predicting the conversion of mild cognitive impairment to Alzheimer’s disease. We implemented a novel multi-modal approach that allowed us to evaluate the potential of different imaging modalities, both alone and in different degrees of combinations, in predicting the conversion to Alzheimer’s disease of mild cognitive impairment patients. We applied this approach to the imaging data from the Alzheimer’s Disease Neuroimaging Initiative that is a multi-modal imaging dataset comprised of MRI, Fluorodeoxyglucose PET, Florbetapir PET and diffusion tensor imaging. We included a total of 480 mild cognitive impairment patients that were split into two groups: converted and stable. Imaging data were segmented into atlas-based regions of interest, from which relevant features were extracted for the different imaging modalities and used to construct machine-learning models to classify mild cognitive impairment patients into converted or stable, using each of the different imaging modalities independently. The models were then combined, using a simple weight fusion ensemble strategy, to evaluate the complementarity of different imaging modalities and their contribution to the prediction accuracy of the models. The single-modality findings revealed that the model, utilizing features extracted from Florbetapir PET, demonstrated the highest performance with a balanced accuracy of 83.51%. Concerning multi-modality models, not all combinations enhanced mild cognitive impairment conversion prediction. Notably, the combination of MRI with Fluorodeoxyglucose PET emerged as the most promising, exhibiting an overall improvement in predictive capabilities, achieving a balanced accuracy of 78.43%. This indicates synergy and complementarity between the two imaging modalities in predicting mild cognitive impairment conversion. These findings suggest that β-amyloid accumulation provides robust predictive capabilities, while the combination of multiple imaging modalities has the potential to surpass certain single-modality approaches. Exploring modality-specific biomarkers, we identified the brainstem as a sensitive biomarker for both MRI and Fluorodeoxyglucose PET modalities, implicating its involvement in early Alzheimer’s pathology. Notably, the corpus callosum and adjacent cortical regions emerged as potential biomarkers, warranting further study into their role in the early stages of Alzheimer’s disease.

## Teaser

Agostinho *et al*. predict conversion to Alzheimer’s in mild cognitive impairment patients. AV45-PET excels as a single modality (83.51%). Combining structural MRI with fluorodeoxyglucose (FDG-PET) yields some promise, showing synergy in terms of improved accuracy (78.43%). These findings underscore the predictive value of β-amyloid markers and reveal modality-specific biomarkers (e.g. brainstem and corpus callosum), urging deeper exploration in early Alzheimer’s pathology.

## Graphical Abstract

**Graphical Abstract:**
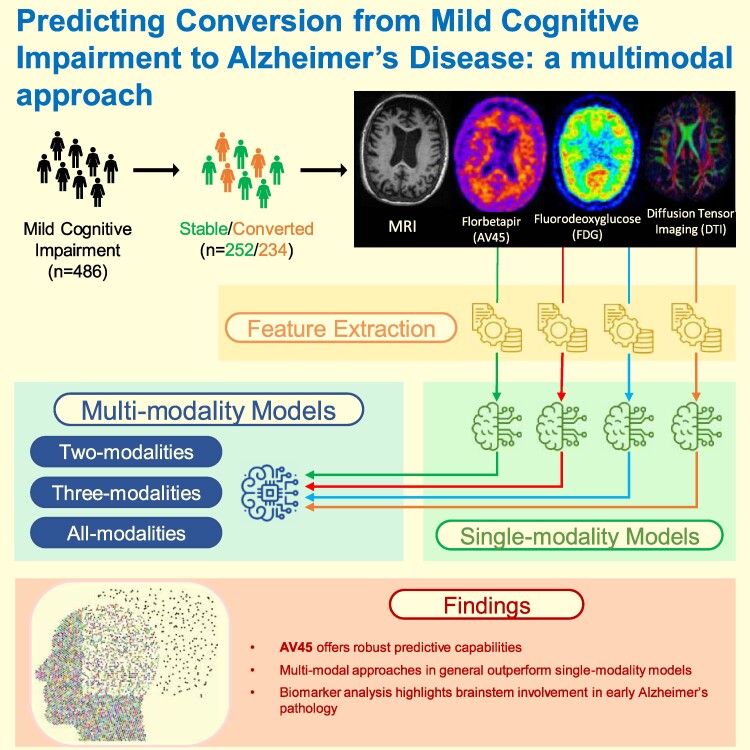

## Introduction

Alzheimer’s disease is a neurodegenerative disorder characterized by progressive brain degeneration and loss of cognitive function, leading to severe dementia and decrease in life expectancy.^1,2^ It is usually diagnosed in people over 65 years of age,^3^ and no cure has yet been found. Despite this, it is anticipated that in the near future treatments capable of not only delaying the advance of symptoms but also slow down the progression of the disease will become available.^4^ Furthermore, it is expected that interventions at earlier stages of the disease can be greatly beneficial. The upcoming treatment options can be tested using tools able to identify individuals likely to progress to Alzheimer’s disease. Thus, the ability to predict whether mild cognitive impairment (MCI) patients will progress to Alzheimer’s disease is of clinical significance, by providing earlier opportunities for diagnosis and allowing to probe more effective treatment options.

Alzheimer’s disease is a progressive disease and before its onset, individuals may experience some cognitive changes that are unexpected for their age and education but that otherwise do not significantly interfere with their daily activities. This prodromal stage can manifest as MCI.^5,6^ Over time, some MCI individuals may convert to Alzheimer’s disease (denoted as converted MCI, cMCI), while others remain stable (defined as stable MCI, sMCI).^7^ Due to the high risk (10–15%) of MCI converting to Alzheimer’s disease,^8-10^ it is very relevant to investigate its progression and development. Furthermore, it is becoming increasingly important to seek effective biomarkers to monitor MCI development, being able to track its progress and predict its eventual conversion to Alzheimer’s disease at an early stage.

The currently available biomarkers for the prediction of MCI conversion to Alzheimer’s disease can be divided into three categories: genetic, chemical and neuroimaging. The latter has been increasingly suggested as powerful not only for the classification of the different stages of Alzheimer’s disease, even surpassing biochemical CSF markers,^11^ but also for predicting the conversion of MCI to Alzheimer’s disease.^12-14^ Tissue volume alterations are commonly used in different classification approaches and can be extracted from MRI. Moreover, the use of PET allows the extraction of measurements such as metabolic alterations or pathological burden, when using appropriate radioactive tracers. Finally, in recent years, diffusion metrics obtained from diffusion tensor imaging (DTI) have been used to detect early white matter (WM) atrophy observed in Alzheimer’s disease.^15^

Data from structural MRI (sMRI) have been widely used to detect structural changes in both early Alzheimer’s disease and MCI.^16-19^ Eskildsen *et al*.^16^ showed that patterns of cortical thickness may be used to identify cortical regions with potential discriminative power for separating sMCI from cMCI. Cheng *et al*.^18^ performed analyses through the use of 93 manually labelled regions of interest (ROIs) and the extraction of the grey matter (GM) tissue volumetric measures for each region, which were afterwards used as features in the classification of the different Alzheimer’s disease stages and prediction of the conversion to dementia in MCI patients.

Fluorodeoxyglucose PET (FDG-PET) allows the measurement of brain glucose metabolism and may therefore provide a sensitive biomarker to differentiation between sMCI and cMCI.^14,20-22^ Caminiti *et al*.^14^ used a multivariate logistic model to identify the best predictors of conversion from MCI to Alzheimer’s disease between FDG-PET and CSF biomarkers. Their findings supported the relevance of FDG-PET biomarkers in predicting progression to Alzheimer’s disease and also showed that these biomarkers outperformed the CSF biomarkers. Blazhenets *et al*.^20^ compared the use of imaging and non-imaging biomarkers to predict the conversion from MCI to Alzheimer’s disease. Their findings suggested that FDG-PET outperforms amyloid PET, but not the non-imaging biomarkers. However, the non-imaging model was improved when adding the FDG-PET data, suggesting that FDG-PET data contain complementary information to the non-imaging biomarkers. In Ottoy *et al*.,^21^ the results showed that neurodegenerative markers from sMRI and FDG-PET were strongly associated with cognitive decline and short-term (1-year) conversion from MCI to Alzheimer’s disease dementia. Mosconi *et al*.^22^ combined genetic and imaging biomarkers and assessed whether this combination would improve prediction of the conversion of MCI to Alzheimer’s disease. The results showed hypometabolism in Alzheimer’s disease-typical regions for the cMCI group that could be used in the improvement of the prediction of conversion of MCI to Alzheimer’s disease.

Moreover, the use of different radioactive tracers, such as Pittsburgh B compound (PiB) or florbetapir (F18-AV45) in PET, allows *in vivo* visualization and quantification of the burden caused by amyloid deposits in the brain.^23,24^ Recently, Florbetapir PET has been used in the study of MCI and its conversion to Alzheimer’s disease.^25-27^ Bouallègue *et al*.^25^ evaluated the value of using amyloid PET textural and shape features in the diagnosis and prognostic evaluation of Alzheimer’s disease. Their results demonstrated that amyloid PET textural and shape analysis yields relevant semi-quantitative markers of amyloid plaque burden, eliminating the need for count ratio methods, such as the ratio between the standard uptake value (SUV) of different regions. In Doraiswamy *et al*.,^26^ Florbetapir PET scans were qualitatively rated as positive or negative amyloid-β (Aβ), according to the tracer retention in different areas. The goal was to evaluate whether subjects with positive scans demonstrated greater cognitive decline than those with negative scans. They found that MCI subjects with positive scans were more likely to convert to Alzheimer’s disease. Ong *et al*.^27^ monitored 45 MCI subjects across 2 years using Florbetapir PET, sMRI and neuropsychological assessments and found that Florbetapir PET can accurately predict which individuals will progress from MCI to Alzheimer’s disease.

DTI is a relatively new yet promising neuroimaging technique with a role still to be defined in the early diagnosis of Alzheimer’s disease as well as in the identification of early at-risk groups, such as MCI patients. Measurements extracted from this imaging modality, such as fractional anisotropy (FA) and mean diffusion (MD), were found to be good indices of fibre density, axonal diameter and myelination and can be useful as early signals of cognitive decline.^28,29^ Gyebnár *et al*.^30^ explored how FA and MD measurements could help in the differentiation of MCI subclasses. Their results showed that these measurements were significantly altered in some regions of the brain and that adding DTI information to volume measurements will help to further identify the disease state.

Each neuroimaging modality captures different aspects of the several stages of the disease. Thus, the combination of biomarkers from different modalities could offer complementary information^31-33^ and, in turn, facilitate a more accurate diagnosis or even prediction of conversion, in comparison to the use of single-modality biomarkers.^34^ In fact, the combination of different modalities has been used in the study of MCI and its conversion to dementia. For example, when combining sMRI, FDG-PET and Florbetapir PET data,^35^ Xu *et al*. obtained superior classification accuracies for predicting the conversion of MCI to Alzheimer’s disease. To establish this comparison, they combined sMRI and FDG-PET (70.6%) and sMRI and Florbetapir PET (64.3%) and analysed the isolated performance of each imaging modality (50.6% for sMRI, 67.0% for FDG-PET and 63.7% for Florbetapir PET). Another example was the combination of FDG-PET and Florbetapir PET data.^36^ Bouallègue *et al*.^36^ found that when analysing the MCI conversion to Alzheimer’s disease paradigm, the combination of data yields the best results (77% accuracy), with higher specificity, accuracy and positive predictive values than FDG-PET (67% accuracy) and Florbetapir PET (72% accuracy) individually.

The primary aim of this study is to investigate the predictive power of different neuroimaging modalities, both individually and in combination, and their ability to successfully differentiate MCI patients into sMCI and patients that afterwards convert from MCI to Alzheimer disease (cMCI). Furthermore, we characterize and evaluate the most sensitive biomarkers extracted from each imaging modality and their impact on the classification paradigm. To achieve it, data from the Alzheimer Disease Neuroimaging Initiative (ADNI) database including four modalities (sMRI, FDG-PET, AV45-PET and DTI) were used. The large number of participants in this dataset allows a proper validation procedure, by deriving independent training and validation sets. Finally, to assure a fair comparison between all modalities, we focus our analysis on atlas-based regions of interest, to identify how different brain regions contribute to each signal modality.

## Materials and methods

### Participants

In this study, we included a total of 486 participants form the ADNI database (adni.loni.usc.edu). The ADNI is a longitudinal multicentre study designed to develop clinical, imaging, genetic and biochemical biomarkers for early detection and tracking of Alzheimer’s disease. For updated information about the project’s status, please refer to adni.loni.usc.edu. The ADNI dataset comprises periodic visits where the participant’s condition is evaluated. At each visit, participants receive a diagnosis, and imaging data from one or more imaging modalities are collected. The number of available modalities varied across these visits.

Participants from ADNI-1 to ADNI-3 and ADNI GO were candidates for inclusion in the current study, and were included whenever they met the following criteria (Supplementary Fig. 1): (i) diagnosed with MCI during their baseline assessment diagnosis; and (ii) they were enrolled in the study for a period of at least 36 months, undertaking periodic assessments of their condition, including clinical diagnosis and at least one of the following imaging modalities (sMRI, FDG-PET, AV45-PET or DTI). The eligible participants were thoroughly inspected and categorized as stable MCI (sMCI; *n* = 252) if they retained the MCI diagnosis throughout all assessments over time, or as converted MCI (cMCI; *n* = 234) if they were diagnosed with Alzheimer’s disease in one of the periodical assessments within the available data and maintained an Alzheimer’s disease diagnosis in all the subsequent assessments. Participants with intermittent diagnosis (MCI to Alzheimer and back to MCI) were discarded. Table 1 summarizes the clinical and demographic information of the included participants, and Supplementary Table 1 provides their IDs in respect to the ADNI database.

**Table 1:** Clinical and demographic characteristics of the subjects included in the study, including age, clinical dementia rating (CDR) and mini-mental state exam (MMSE) for both the beginning and end timepoint of data collection

	sMCI	cMCI	*P*-value
Gender	104F/148M	87F/147M	0.35
Initial age	72.42 ± 7.44	74.20 ± 6.94	<0.01
Final age	76.94 ± 7.75	76.34 ± 7.22	0.38
Initial CDR	0.50 ± 0.00	0.50 ± 0.00	N/A
Final CDR	0.53 ± 0.21	0.55 ± 0.17	0.18
Initial MMSE	27.99 ± 1.71	27.07 ± 1.70	<0.01
Final MMSE	27.29 ± 3.17	25.90 ± 2.26	<0.01
Time of conversion (months)		35.03 ± 26.51	

Data are represented as mean ± standard deviation.

To ensure a fair validation process, eligible participants categorized above as cMCI and sMCI were divided into two sets: the Incomplete Visits Set (IVS) and the Complete Visits Set (CVS). This separation was determined according to the following criteria: participants possessing multi-modal imaging data from all four imaging modalities of interest (sMRI, FDG-PET, AV45-PET and DTI) in at least one assessment were included in the CVS. Participants lacking data from at least one of the imaging modalities in all the visits were included in the IVS (refer to Supplementary Fig. 2).

This division was due to most participants’ visits in the dataset being incomplete regarding the registered imaging modalities, and due to our multi-modality approach being based on an ensemble of single-modality classifiers (as explained below). While complete multi-modality examples (visits) are necessary to validate the ensemble models, they are not required to train the single-modality models, which can be trained in independent single-modality datasets. This division ensures a fair comparison between the multi-modality model and the single-modality models, preserving data from participants with complete visits for model validation. Ultimately, the CVS consisted of 92 subjects (27 cMCI and 65 sMCI), with a total of 127 sets of full scans (32 cMCI and 95 sMCI). The IVS varied in size for each imaging modality, and a comprehensive breakdown of all participants and modalities can be found in Supplementary Table 2.

### Data analysis

sMRI data were processed and analysed using the Computational Anatomy Toolbox (CAT12; http://www.neuro.uni-jena.de/cat). The CAT12 toolbox runs on Statistical Parametric Mapping (SPM12; Welcome Department of Imaging Neuroscience, London, UK) implemented in MATLAB R2020a (The MathWorks Inc., Natick, MA, USA). The structural images were ﬁrst corrected for bias, noise and global intensity; corrected images were spatially normalized to the MNI152 template, using the DARTEL algorithm.^37^ The normalized images were segmented into GM, WM and CSF^38,39^ and the absolute volume of each tissue determined by multiplying the voxel values with the Jacobian determinant derived during spatial normalization. Total intracranial volume (TIV) was calculated for each individual during segmentation and was used to control for head size in subsequent analysis. Finally, all scans were smoothed with a 6 mm Gaussian kernel and resampled into 1.5 mm^3^ voxel size. Quality assurance was conducted via visual inspection and an automated quality check protocol embedded in CAT12. The pre-processed data were then analysed using region-based morphometry. In this approach, an anatomical atlas is transformed into the native subject space and the sum of the local tissues inside the atlas’ pre-defined ROIs is estimated. The Hammers atlas^40,41^ was chosen, providing a total of 64 ROIs (Supplementary Fig. 3). For each ROI, the mean value of GM and WM was extracted and normalized for the TIV.

Both FDG-PET and AV45-PET data were processed and analysed using the Statistical Parametric Mapping (SPM12; Welcome Department of Imaging Neuroscience, London, UK) implemented in MATLAB R2020a (The MathWorks Inc., Natick, MA, USA). The functional images were initially summed for the total duration of the acquisition. The summed images were then normalized to the ICBM152 space. The normalized images were then visually inspected to ensure that no obvious imperfections occurred during the normalization process. The images were smoothed using a Gaussian kernel with full width at half maximum (FWHM) of 8 mm. The pre-processed functional images were analysed in a similar way as the structural data using the Hammers atlas^40,41^ plus four reference regions (cerebellum, brain grey matter, brain white matter and pons). For each ROI of the Hammers atlas, the mean activation value was extracted and used as a feature by calculating the standard uptake value ratio (SUVR) for the different reference regions. In the end, four different feature sets were constructed, which will be referred, from now on, as cerebellum-, GM-, WM- and pons-based features (Supplementary Fig. 3).

DTI data were processed using the ExploreDTI diffusion MRI toolbox (https://www.ExploreDTI.com),^42^ and processing the data comprised several steps. First, the scans were corrected for eddy current, induced geometrical distortions and echo planar imaging (EPI) deformations, setting the optional parameters of deformation axes to [1 0 0] and image type to FA.^43,44^ Following this step, the corrected DTI scans were spatially normalized for the HCP-1065 diffusion MRI template.^45^ As with the previous imaging modalities, the data were analysed using the Hammers atlas^40,41^ and for each ROI the mean diffusion metrics FA and MD were extracted (Supplementary Fig. 3).

### Single-modality classification

To predict the conversion of MCI to Alzheimer’s disease, we implemented a supervised machine-learning approach. Support vector machine (SVM) models were constructed for each neuroimaging modality. To effectively train and validate each of these models, the available data were split into a training and test group. Given the specific characteristics of our experimental design, the data within the test group must be exclusively derived from participants in the CVS. This is crucial for later comparison of the effects of different ensemble single-modality classifiers, requiring that the test group participants possess visits with all four imaging modalities. So, we split the data into training and testing in a way that the training data incorporate all the data from participants in the IVS and 30% of randomly selected participants from the CVS. Conversely, the test data comprise the remaining 70% of the CVS participants’ data. This way, train set comprises ∼73.86% of the total data and the test set the remainder 26.14%.

Following the division of data into the training and test groups, the data in the training group were employed to train and establish the SVM models. Leveraging the scikit-learn API,^46^ we constructed the SVM models, setting the gamma parameter to ‘scale’ to address class imbalance and determining the optimal *c*-value in a 10-fold cross-validation setting. Notably, the ‘scale’ setting within the scikit-learn API automatically computes the ideal gamma value based on Eq. (1).


(1) $$\begin{array}{r} \mathrm{gamma}=\;\frac{1}{\mathrm{number}\;\mathrm{of}\;\mathrm{features}\;*\;\mathrm{variance}(\mathrm{training}\;\mathrm{data})\;} \end{array}.$$

The optimal *c*-value was estimated using the ‘optuna’ optimization framework,^47^ where the candidate *c*-values are derived from an iterative Bayesian optimization procedure. For each *c* candidate, a 10-fold cross-validation method was used to evaluate the hyperparameter performance. The training data (9-folds) were normalized to have zero mean and unit variance, and the mean and standard deviation of the training data were used to normalize the validation data (1-fold). The final performance of each candidate *c*-value was derived as the mean balanced accuracy across all the folds. In the end, the *c*-value that produced the best balanced accuracy across folds was selected for the final model. This *c*-value was then used to construct the SVM model using all the training data. Two different kernels were tested: linear and the radial basis function (RBF). The model’s performance was evaluated against the test data, and the metrics of balanced accuracy, sensitivity, specificity, F1-score, negative predictive value (NPV) and positive predictive value (PPV) were extracted (Supplementary Fig. 4). Additionally, to validate the statistical significance of each model’s balanced accuracy, we performed a permutation test and corrected its significance for multiple comparisons using the restrictive Bonferroni method. This process was repeated 30 times, each time with a new random split for constructing the train and test sets, and the final model’s performance was derived from the mean of all the metrics.

### Multi-modality classification

To evaluate the effects of combining the different imaging modalities, an ensemble framework was adapted. Here, for each of the 30 repetitions, the best classifiers from each imaging modality were combined using a non-generative weighted fusion technique. The final prediction decision is the result of the combination of the weighted base classifier’s predicted probabilities. This fusion is achieved by assigning a weight value (*c_k_*) between 0 and 1 to each base classifier *k* to calculate the final ensemble prediction probability (*Y_i_*) as shown in Eq. (2).


(2) $$Y_{i}=\;\sum_{k}c_{k}y_{ki}.$$

Here, *Y_i_* stands for the final ensemble prediction probability for the case *i*, *y_ki_* stands for the predicted probability of the *k* base classifier for the case *i*, and the *c_k_* corresponds to the weight applied to the *k* base classifier, so that $\sum_{k}c_{k}=1$. The weights (*c_k_*) were equally assigned to all the modalities to assure no bias was given to any imaging modality. In the end, the ensemble model’s performance was determined by taking the mean performance across the 30 different repetitions.

### Classification using the subset of the most relevant biomarkers

We evaluated the relative importance of each neuroimaging biomarker for the classification task in each of the selected models from the ‘single-modality classification’. In this context, each ROI from the Hammers atlas serves as a feature used to build the classification models and can be considered as an individual neuroimaging biomarker. We conducted a two-step analysis to determine the optimal number of features for building each classification model and to evaluate the importance of each biomarker.

For each model, corresponding to each imaging modality, we followed a similar approach as when developing the single-modality classifiers, randomly splitting the participants into training and test sets following the approach previously described. Subsequently, we used the training data to rank each ROI based on their relative importance scores calculated using the minimum-redundancy-maximum-relevance (mRMR) feature selection method, implemented in the Python environment^48^ using the mrmr-selection module. This method ranks each biomarker according to its relevance while minimizing redundancy between features. The ranked features represent the most relevant biomarkers, and their influence on the classification problem was evaluated in the following step.

Using the previously determined ranks, we iteratively generated new classification models containing subsets of the total number of ranked features, starting only with the best ranked feature and, iteratively, adding the next highestly ranked feature. For example, if the ranked features are denoted as [f1, f2, …, f64], the first new model was constructed using only f1, the second using [f1 and f2], and so on until the subset included the full set. For each feature set, an optimal *c*-value was derived using the ‘optuna’ optimization method, employing the same 10-fold cross-validation as with the single-modality classifiers. Ultimately, for each feature set, the mean balanced accuracy across all folds was calculated and used to represent its performance.

To select the best subset of features (i.e. the best model), we used a derivation of the Bayesian information criterion (BIC) given by


(3) $$\mathrm{BIC}=n\;\log\frac{L^{2}}{n}+k\;\log\;n$$

where *n* represents the number of samples, *L* is the loss of the model derived from the mean balanced accuracy (100 − balanced accuracy), and *k* is the number of features used in the model. This metric optimizes for the lowest error while penalizing the complexity of the models, promoting models with less features following the parsimony principle. So, for each feature set, we computed the BIC and selected the subset with the lowest score as the best model. We then used the selected subset and the respective obtained *c*-value to construct the final model and evaluated it against the data from the test group.

Similar to the single-modality classifiers, this approach was repeated 30 times, and the feature ranking, optimal number of features and model performance were calculated using the mean across all repetitions. Additionally, different ensemble multi-modality combinations were evaluated for each repetition.

## Results

### Single-modality classification

The performance results from the classifiers for the sMRI, AV45-PET, FDG-PET and DTI imaging modalities are summarized in Table 2. All the presented models were constructed using 64 features, one from each ROI of the Hammers atlas, extracted from the different feature maps (e.g. sMRI-GM and FDG-WM) (Supplementary Fig. 3). The mean optimal *c*-value estimated for each model can be found in Supplementary Table 3.

**Table 2:** Performance of the single-modality classifiers

Modality	Feature	Kernel	Acc	Sen	Spe	F1-score	NPV	PPV	AUC	*P*-value
sMRI	**GM**	Linear	72.52 ± 0.54	67.25 ± 0.83	77.78 ± 0.59	0.76 ± 0.00	87.65 ± 0.32	50.50 ± 0.82	0.76 ± 0.01	<0.01
		**RBF**	**72.75** ± **0.78**	**66.69** ± **1.34**	**78.81** ± **0.80**	**0.77** ± **0.01**	**87.64** ± **0.47**	**51.60** ± **1.02**	**0.80** ± **0.01**	**<0**.**01**
	WM	Linear	55.45 ± 0.69	28.80 ± 1.46	82.11 ± 0.72	0.68 ± 0.01	77.56 ± 0.38	34.86 ± 1.17	0.61 ± 0.01	0.13 ± 0.02
		RBF	58.52 ± 0.87	42.16 ± 1.78	74.88 ± 0.80	0.67 ± 0.01	79.55 ± 0.51	35.89 ± 1.18	0.60 ± 0.01	0.09 ± 0.03
AV45	**Cerebellum**	**Linear**	**83.51** ± **0.59**	**85.83** ± **0.81**	**81.19** ± **0.75**	**0.83** ± **0.01**	**94.48** ± **0.33**	**60.80** ± **0.98**	**0.86** ± **0.00**	**<0**.**01**
		RBF	79.41 ± 0.62	78.23 ± 0.97	80.59 ± 0.86	0.81 ± 0.01	91.74 ± 0.35	57.93 ± 1.06	0.85 ± 0.00	<0.01
	GM	Linear	83.39 ± 0.44	87.76 ± 0.70	79.01 ± 0.76	0.82 ± 0.00	95.10 ± 0.28	58.67 ± 0.87	0.86 ± 0.00	<0.01
		RBF	78.22 ± 0.87	76.78 ± 1.37	79.67 ± 0.80	0.80 ± 0.01	91.11 ± 0.53	56.16 ± 1.17	0.84 ± 0.01	<0.01
	WM	Linear	82.06 ± 0.46	87.60 ± 0.67	76.53 ± 0.84	0.81 ± 0.01	94.88 ± 0.27	55.89 ± 0.85	0.86 ± 0.00	<0.01
		RBF	80.20 ± 0.40	81.45 ± 1.01	78.96 ± 0.92	0.81 ± 0.00	92.82 ± 0.33	57.00 ± 0.88	0.85 ± 0.00	<0.01
	Pons	Linear	82.41 ± 0.47	83.40 ± 0.83	81.42 ± 0.68	0.83 ± 0.00	93.66 ± 0.29	60.34 ± 0.85	0.85 ± 0.00	<0.01
		RBF	80.36 ± 0.75	79.29 ± 1.11	81.43 ± 0.85	0.82 ± 0.01	92.18 ± 0.42	59.32 ± 1.23	0.86 ± 0.00	<0.01
FDG	Cerebellum	Linear	68.21 ± 0.73	68.30 ± 1.41	68.11 ± 0.84	0.70 ± 0.01	86.59 ± 0.56	41.86 ± 0.65	0.75 ± 0.01	<0.01
		RBF	64.17 ± 0.73	59.55 ± 1.62	68.80 ± 0.90	0.68 ± 0.01	83.64 ± 0.57	39.05 ± 0.67	0.71 ± 0.01	<0.01
	**GM**	Linear	69.02 ± 0.82	70.92 ± 1.31	67.12 ± 0.87	0.70 ± 0.01	87.35 ± 0.57	42.08 ± 0.79	0.78 ± 0.01	<0.01
		**RBF**	**71.39** ± **0.65**	**69.17** ± **1.14**	**73.60** ± **0.90**	**0.74** ± **0.01**	**87.74** ± **0.43**	**47.05** ± **0.90**	**0.76** ± **0.01**	**<0**.**01**
	WM	Linear	69.16 ± 0.75	68.13 ± 1.73	70.19 ± 0.98	0.71 ± 0.01	86.96 ± 0.59	43.53 ± 0.70	0.78 ± 0.01	<0.01
		RBF	69.63 ± 0.75	67.66 ± 1.09	71.60 ± 0.89	0.72 ± 0.01	86.87 ± 0.44	44.68 ± 1.00	0.74 ± 0.01	<0.01
	Pons	Linear	68.84 ± 0.71	76.77 ± 1.15	60.92 ± 0.82	0.67 ± 0.01	88.74 ± 0.52	39.76 ± 0.66	0.75 ± 0.01	<0.01
		RBF	64.61 ± 0.76	68.93 ± 1.36	60.30 ± 0.79	0.65 ± 0.01	85.37 ± 0.60	36.79 ± 0.64	0.76 ± 0.01	<0.01
DTI	FA	Linear	56.07 ± 1.21	55.40 ± 3.37	56.74 ± 2.56	0.58 ± 0.01	79.88 ± 1.05	30.17 ± 1.02	0.59 ± 0.01	0.20 ± 0.04
		RBF	52.51 ± 0.84	36.02 ± 3.17	69.00 ± 3.18	0.61 ± 0.02	76.35 ± 0.53	29.06 ± 1.64	0.56 ± 0.01	0.28 ± 0.04
	**MD**	Linear	56.22 ± 0.85	53.02 ± 2.23	59.42 ± 1.90	0.60 ± 0.01	79.21 ± 0.61	30.75 ± 0.91	0.56 ± 0.01	0.15 ± 0.03
		**RBF**	**57.77** ± **1.10**	**60.43** ± **2.96**	**55.11** ± **2.12**	**0.59** ± **0.01**	**81.19** ± **0.88**	**31.08** ± **0.96**	**0.59** ± **0.01**	**0.14 ± 0.03**

The best models for each imaging modality are highlighted in bold. Acc, balance accuracy; Sen, sensitivity; Spe, specificity; NPV, negative predictive value; PPV, positive predictive value; AUC, area under the curve.

**Figure 1:**
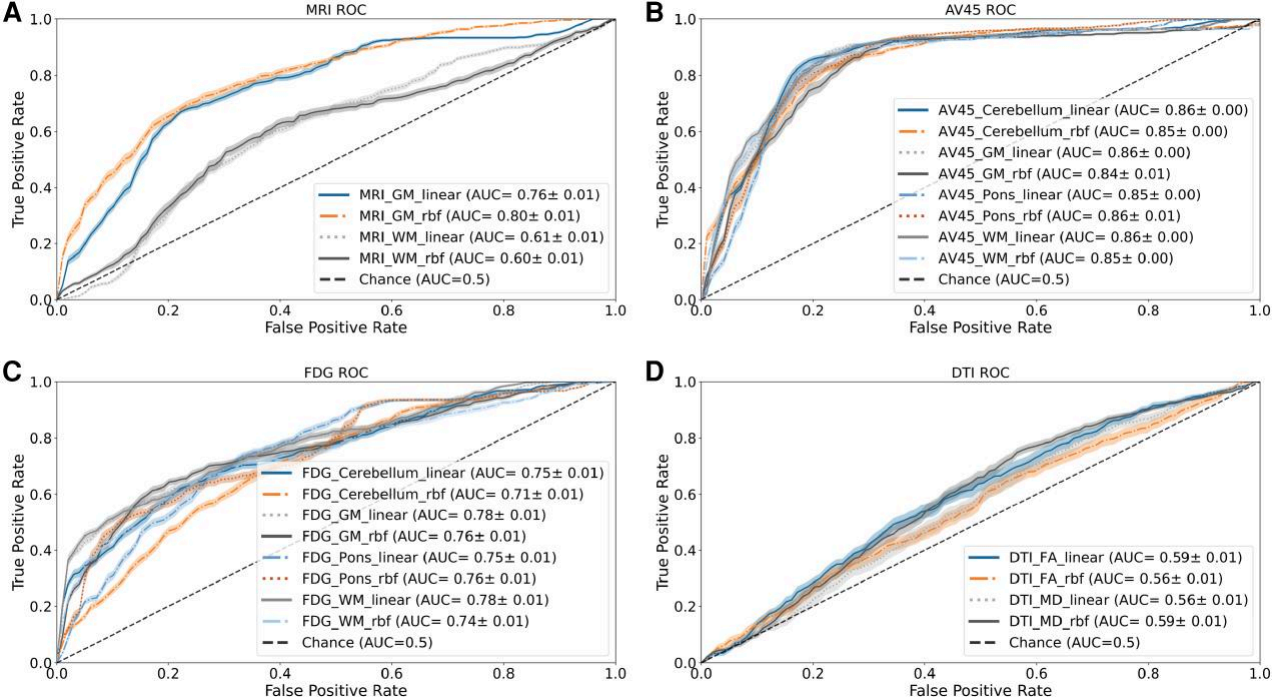
**Receiver operating characteristic (ROC) curve for all the single-modality models developed.** The figure presents the ROC curves and their respective area under the curve (AUC) for all the models created using different imaging modalities. The ROC curves provide a comprehensive visual representation of the performance of each single-modality model. The AUC values offer a quantitative measure of the models’ overall accuracy, serving as an essential metric for evaluating their predictive performance. (**A**) ROC curve for all the structural magnetic resonance imaging (sMRI) based models, which utilized white matter (WM) and grey matter (GM) features and employed both linear and radial basis function (rbf) kernels; (**B**) ROC curve for all the florbetapir (AV45) based models, which utilized white matter (WM), grey matter (GM), pons and cerebellum as features and used both linear and radial rbf kernels; (**C**) ROC curve for all the fluorodeoxyglucose (FDG) based models, which utilized white matter (WM), grey matter (GM), pons and cerebellum as features and employed both linear and radial rbf kernels; (**D**) ROC curve for all the diffusion tensor imaging (DTI) based models, which utilized fractional anisotropy (FA) and mean diffusion (MD) features and used both linear and rbf kernels.

Regarding the sMRI-based models, GM-based features vastly outperformed WM-based features. The models built using GM-based features achieved a mean balanced accuracy of around 72%, while those built using WM-based features achieved a maximum balanced accuracy of around 58%. When comparing the two SVM kernels, it is evident that, for both feature maps (GM and WM), the RBF kernel outperforms the linear kernel. This difference is more noticeable in the models using WM-based features, where the balanced accuracy of the model improves by 3% when comparing the two kernels. Focusing on the best models from this imaging modality, which are built using GM-based features, both exhibit remarkable specificity, reaching mean values between 77% and 79%. They also achieve high values of NPV, reaching 87.65%. However, both the sensitivity and PPV of these models are low, with a maximum mean value of 67.25% and 51.60%, respectively. Furthermore, Fig. 1A shows the receiver operating characteristic (ROC) curve for all models and the respective area under the curves (AUCs). Observing the figure, it is possible to see that the GM-based models have AUC values of 0.76 and 0.80 for the linear and RBF kernel, respectively, outperforming the WM-based models that reached AUC values around 0.60. Ultimately, the best overall model for the sMRI imaging modality was achieved using the RBF kernel and GM-based features with a mean accuracy of 72.75%.

AV45-PET-based classifiers exhibited the highest overall performance among all the studied imaging modalities. When comparing the different reference regions, it is evident that they demonstrated similar performance levels, with mean balanced accuracy values ranging between 78% and 84%. In contrast to the sMRI models, the utilization of the linear kernel proved to be more advantageous than the RBF non-linear kernel in AV45-PET models. This resulted in improvements of at least 2% (e.g. when using the pons as the reference feature map) and up to 5% (e.g. when using the GM as the reference feature map) for specific feature maps. Similar to the top-performing sMRI models, these AV45-PET models displayed remarkable values of mean specificity (ranging from 76.53% to 81.43%) and NPV (ranging from 91.11% to 94.88%). Additionally, in contrast to the previous models, they also demonstrated excellent mean sensitivity values, ranging from 78.22% to 87.76%. However, these models still reported relatively low values of PPV, with a maximum mean value of 60.80%. As with the sMRI models, we also extracted the ROC curve and the respective AUC for each model. These results are shown in Fig. 1B, where we can observe that the ROC curves of all models are clustered together, confirming that these models have similar performance, reaching AUC values around 0.86. Overall, the best AV45 model was achieved using the linear kernel and cerebellum-based features, with the best overall mean accuracy of 83.21%.

The FDG-PET models do not follow a straightforward tendency, as observed in the previous two imaging modalities. When comparing the results of the different reference regions, they show similar performance, with the exception of the model built using the RBF kernel and GM-based features, which achieved the lowest balanced accuracy with a mean value of 64.17%. This value is significantly lower than the rest of the models, whose mean balanced accuracy varies between 68% and 72%. Regarding the kernel used, there is no clear advantage to using one over the other. The linear kernel outperformed the RBF kernel when using the pons- and cerebellum-based features, while the reverse trend was observed when using the other feature maps. However, in the higher-performing models using GM- and WM-based features, the non-linear RBF kernel outperformed the linear kernel. Furthermore, similar to the previous models from other imaging modalities, we observe high values of mean specificity, sensitivity and NPV, reaching mean values as high as 73.60%, 76.77% and 87.74%, respectively. The ROC curves for these models (Fig. 1C) show a similar dispersion as the AV45-PET models, with all the models clustered together. However, contrary to the previous PET modality, there seems to be a performance discrepancy that is mainly visible in the beginning of the ROC curve. In the end, these models manage to reach AUC values between 0.71 and 0.78. Using this imaging modality, the best model was achieved using the RBF kernel and GM-based features, reaching a mean balanced accuracy of 71.39%.

Finally, the DTI-based models performed the poorest among all the imaging modalities and were unable to consistently outperform chance levels. Both FA- and MD-based feature models exhibited low accuracy values, with MD-based features outperforming FA-based features. This difference was particularly noticeable when using the non-linear RBF kernel, where the gap between the two feature sets was ∼5%. All DTI-based models demonstrated similar performance, with a mean balanced accuracy value of around 56–57%, except for the model built using the linear kernel and FA-based features, which achieved a mean balanced accuracy of 52.51%. Furthermore, these models displayed relatively low values across all performance metrics, except for NPV, which followed a similar trend to the models from other imaging modalities, reaching a mean value as high as 81.19%. These models showed the worst ROC curves, which were close to the chance line. Despite this, all models’ ROC curves were clustered together and above the chance line, reaching AUC values that varied between 0.56 and 0.59. The best overall model for the DTI imaging modality was achieved using the RBF kernel and MD-based features, resulting in a mean balanced accuracy of 57.77%.

In conclusion, among all the single-modality models, those derived from AV45-PET demonstrated the best overall performance, followed by FDG-PET and sMRI, with the DTI-based models performing the poorest.

### Multi-modality classification

After evaluating each imaging modality individually, the best-performing models were selected for multi-modality ensemble classifications. The chosen models where the following sMRI-GM-RBF, AV45-PET-Cerebellum-Linear, FDG-PET-GM-RBF and the DTI-MD-RBF, each one representing one imaging modality. From this point forward, each model will be referred to by its corresponding imaging modality name.

**Figure 2:**
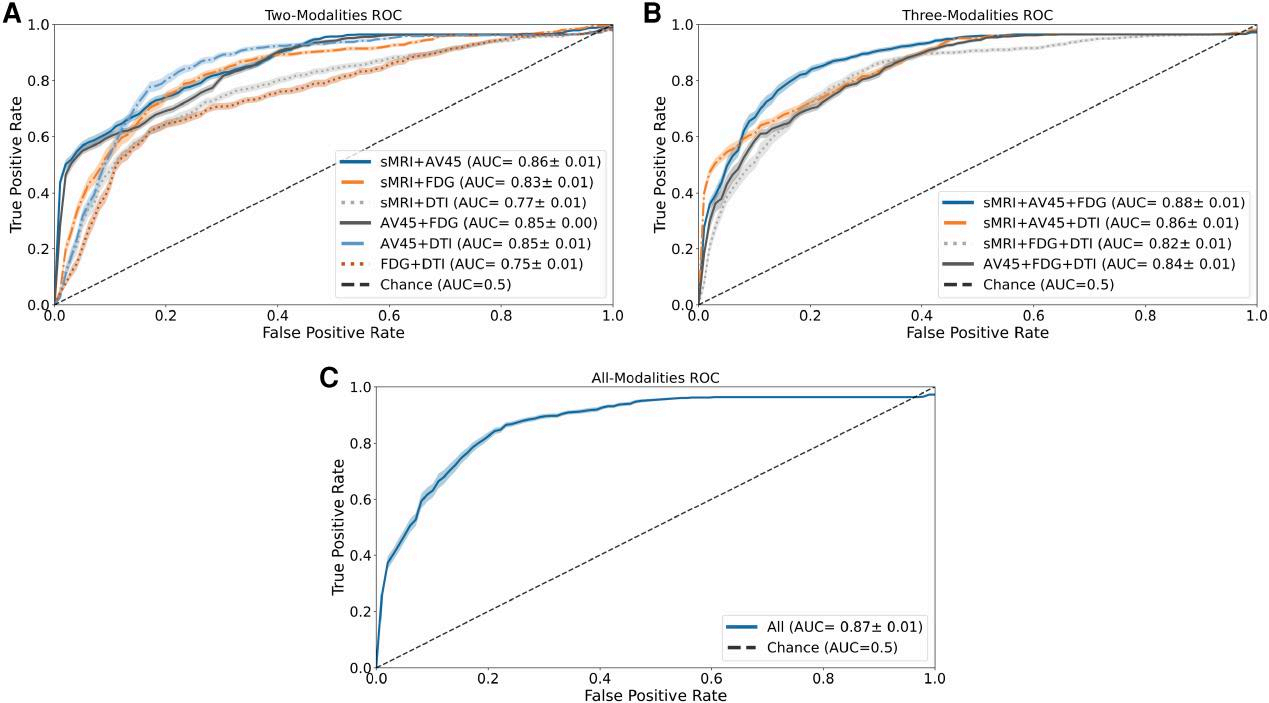
**Receiver operating characteristic (ROC) curve for all the multi-modality models developed.** The figure illustrates the ROC curves and their respective area under the curve (AUC) values for the various combinations of the four neuroimaging modalities included in the study: structural magnetic resonance (sMRI), fluorodeoxyglucose (FDG), florbetapir (AV45) positron emission tomography (PET) and diffusion tensor imaging (DTI). (**A**) Two-modality combinations: this section displays the ROC curves for the models that were constructed by combining two different imaging modalities. Each curve represents the performance of a specific combination, showcasing the discrimination capabilities of the two modalities used together. (**B**) Three-modality combinations: in this part of the figure, the ROC curves for the models created through the combination of three imaging modalities are presented. Each curve represents a specific combination, revealing how the inclusion of an additional modality impacts the model’s predictive performance. (**C**) All modality combinations: this section shows the ROC curve for the model constructed by combining all four imaging modalities.

A total of eleven different combinations were evaluated, starting with a dual-modality approach, followed by a triple-modality approach and finally combining all available modalities. In each combination, equal weights were assigned to classifiers across all imaging modalities to ensure unbiased predictions. The performance of the multi-model classifiers is summarized in Table 3, and the respective ROC curves can be found in Fig. 2.

**Table 3:** Results from all the multi-modal analysis

Combination	Balance accuracy	Sensitivity	Specificity	F1-score	NPV	PPV	AUC	*P*-value
**sMRI + AV45**	76.02 ± 0.77	65.35 ± 1.47	86.69 ± 0.74	0.81 ± 0.01	88.26 ± 0.44	62.88 ± 1.38	0.86 ± 0.01	<0.01
**sMRI + FDG**	**78.43** ± **0.77**	**77.12** ± **1.07**	**79.75** ± **0.86**	**0.80** ± **0.01**	**91.23** ± **0.40**	**56.53** ± **1.24**	**0.83** ± **0.01**	**<0**.**01**
**sMRI + DTI**	71.42 ± 0.75	58.11 ± 1.41	84.74 ± 0.76	0.78 ± 0.01	85.86 ± 0.40	56.62 ± 1.26	0.77 ± 0.01	<0.01
**AV45 + FDG**	74.67 ± 0.71	62.71 ± 1.22	86.63 ± 0.97	0.81 ± 0.01	87.44 ± 0.38	62.54 ± 1.91	0.85 ± 0.01	<0.01
**AV45 + DTI**	52.27 ± 0.42	6.99 ± 1.00	97.55 ± 0.32	0.67 ± 0.01	75.84 ± 0.29	47.24 ± 5.29	0.85 ± 0.00	1.02 ± 0.25
**FDG + DTI**	71.12 ± 0.62	55.79 ± 1.30	86.46 ± 0.81	0.79 ± 0.01	85.44 ± 0.37	58.78 ± 1.30	0.75 ± 0.01	<0.01
**sMRI + AV45 + FDG**	78.79 ± 0.84	68.46 ± 1.31	89.12 ± 0.69	0.84 ± 0.01	89.40 ± 0.46	68.41 ± 1.52	0.88 ± 0.01	<0.01
**sMRI + AV45 + DTI**	73.24 ± 0.70	48.88 ± 1.43	97.61 ± 0.49	0.84 ± 0.01	85.14 ± 0.36	88.85 ± 1.91	0.86 ± 0.01	<0.01
**sMRI + FDG + DTI**	72.53 ± 0.92	57.33 ± 1.48	87.73 ± 0.77	0.80 ± 0.01	86.00 ± 0.48	61.70 ± 1.79	0.82 ± 0.01	<0.01
**AV45 + FDG + DTI**	69.29 ± 0.64	43.70 ± 1.35	94.88 ± 0.58	0.80 ± 0.00	83.48 ± 0.36	76.01 ± 2.17	0.84 ± 0.01	<0.01
**All**	71.94 ± 0.77	50.57 ± 1.50	93.31 ± 0.54	0.81 ± 0.01	85.00 ± 0.41	72.54 ± 1.79	0.87 ± 0.01	<0.01

The combinations that showed an improvement in the classification ability are highlighted in bold.

Regarding the two-modality approach, six combinations were evaluated. Only one combination showed improvement over individual base classifiers. The combination of sMRI with FDG-PET demonstrated enhanced classification ability, improving all classification metrics and achieving a mean balanced accuracy of 78.43%. This result corresponds to a performance improvement of around 5% and 7% compared to the individual classifiers. All combinations showed an improvement in specificity at the cost of a decrease in sensitivity, except for the combination of sMRI with FDG-PET. It is also noteworthy that, excluding the combinations where AV45-PET is present, there was an increase in the F1-score for those combinations and an overall improvement in the PPV for all combinations. Finally, by observing Fig. 2A, it is possible to observe that there appear to be two clusters of models. One cluster is closer to the chance line and encompasses the combination of FDG-PET with DTI and sMRI with DTI, with AUC values of 0.75 and 0.77, respectively. The other cluster contains the remaining two-modality combinations, with AUC values ranging from 0.83 to 0.86.

In the three-modality approach, four combinations were evaluated. However, none of these combinations demonstrated an overall performance improvement over the individual base classifiers. Nevertheless, it is worth noting that the combination of three imaging modalities produced similar effects to the combination of two modalities. Specifically, we observed an improvement in specificity and PPV in all models, as well as an increase in the F1-score for all combinations except for the combination of AV45-PET with FDG-PET and DTI. Additionally, similar to the two-modality combinations, there was a decrease in the models’ sensitivity across all combinations. The ROC curve analysis for these combinations showed a single cluster containing all the different combinations with AUC values between 0.82 and 0.88, which were achieved by the combination of sMRI with FDG-PET and DTI, and sMRI with AV45-PET and FDG, respectively. These curves are similar to the best-performing ROC curves from the two-modality combinations, indicating a lack of improvement by adding a new modality.

Finally, the combination of all base classifiers resulted in a performance similar to that of the sMRI base classifier, with a mean balanced accuracy of 71.94%. This performance was lower compared to most of the other combinations. However, it is noteworthy that even in this case, we observed an improvement in both specificity and PPV, along with a decrease in sensitivity. These patterns align with the trends observed in earlier combinations. Furthermore, we did not observe any improvement in the ROC curve, which achieved an AUC value of 0.87, falling within the same range as the previous models.

### Classification using the subset of the most relevant biomarkers

**Figure 3:**
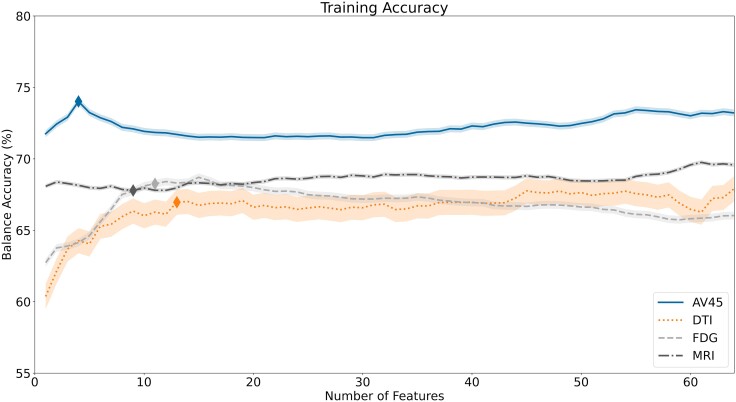
**Training accuracy obtained during the selection of the optimal number of sensitive features.** The figure presents the mean training accuracy achieved for each selected model from the respective imaging modalities, namely structural magnetic resonance (sMRI), fluorodeoxyglucose (FDG), florbetapir (AV45) positron emission tomography (PET) and diffusion tensor imaging (DTI). The training accuracy is shown across different scenarios, where the number of sensitive biomarkers used in the models varies. The optimal set of features was extracted by estimated the mean training accuracy and respective standard error using the different sets of features for each imaging model. Then, the Bayesian information criterion (BIC) score was employed to evaluate the trade-off between model complexity and performance, determining the optimal number of features. The optimal number of sensitive features obtained was 9, 4, 11 and 13 for the sMRI, AV45-PET, FDG-PET and DTI, respectively (see diamond shapes in figure).

**Figure 4:**
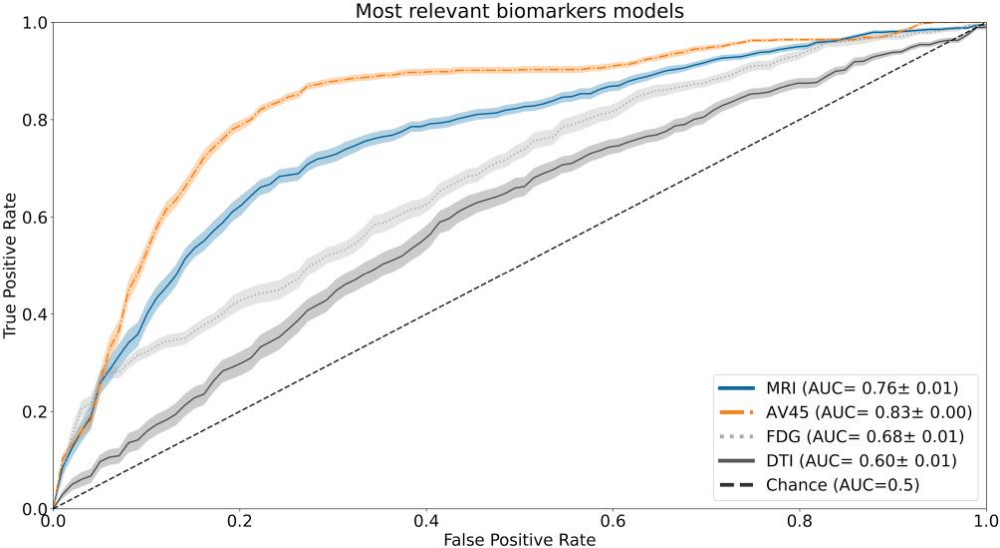
**Receiver operating characteristic (ROC) curve for the single-modality models developed using the most sensitive biomarkers.** The ROC curve illustrates the performance of the different neuroimaging modalities included in this study, namely structural magnetic resonance imaging (sMRI), fluorodeoxyglucose positron emission tomography (FDG-PET), florbetapir positron emission tomography (AV45-PET) and diffusion tensor imaging (DTI). The models were constructed using the identified most sensitive biomarkers for each modality. The area under the curve (AUC) values are provided to quantitatively measure the discriminative power of each model.

Lastly, we conducted an analysis to assess the importance of each neuroimaging biomarker in the classification task for the selected models. Using the mRMR method, we managed to rank each neuroimaging biomarker (see Supplementary Table 4) and summarized the top 10 most relevant biomarkers for each imaging modality in Supplementary Table 5. This ranking was then utilized to build new models, incrementally increasing the number of features. To select the optimal set of features, we extracted the mean training accuracy and respective standard error using the different sets of features for each imaging model (Fig. 3). Then, the BIC score was employed to evaluate the trade-off between model complexity and performance, determining the optimal number of features. The optimal number of features, as well as the new model’s performance, is summarized in Table 4, and the model’s respective ROC curves can be found in Fig. 4. The analyses in Table 4 show no improvement in the overall classification performance for all models. Despite this, both the sMRI and DTI models, built using a vastly inferior number of features, manage to retain almost all of their classification performance. Regarding the new models’ ROC curves, they are similar to the all-features models.

**Table 4:** Performance of the single-modality models using the most sensitive biomarkers (top) as well as with all features (bottom)

Model	#Features	*c*-Value	Balance accuracy	Sensitivity	Specificity	F1-score	NPV	PPV	AUC	*P*-value
Most relevant biomarkers										
sMRI	9 ± 2	0.678 ± 0.232	71.47 ± 0.69	69.20 ± 0.96	73.72 ± 0.99	0.74 ± 0.01	87.54 ± 0.38	47.76 ± 1.01	0.76 ± 0.01	<0.01
AV45	4 ± 1	0.01 ± 0.003	78.99 ± 0.51	76.66 ± 0.93	81.32 ± 0.57	0.82 ± 0.01	91.20 ± 0.33	58.24 ± 0.81	0.83 ± 0.00	<0.01
FDG	11 ± 1	0.19 ± 0.08	62.24 ± 0.80	69.13 ± 1.48	55.36 ± 1.14	0.61 ± 0.01	84.34 ± 0.566	34.37 ± 0.76	0.68 ± 0.01	0.04 ± 0.01
DTI	3 ± 1	299.99 ± 75.96	56.99 ± 0.84	67.36 ± 2.87	46.63 ± 3.19	0.53 ± 0.02	82.05 ± 1.04	30.35 ± 0.715	0.60 ± 0.01	0.12 ± 0.03
All features										
sMRI	64	1.3E^2^ ± 0.3E^2^	72.75 ± 0.78	66.69 ± 1.34	78.81 ± 0.80	0.77 ± 0.01	87.64 ± 0.47	51.60 ± 1.02	0.80 ± 0.01	<0.01
AV45	64	1.1E^−2^ ± 3.7E^−4^	83.51 ± 0.59	85.83 ± 0.81	81.19 ± 0.75	0.83 ± 0.01	94.48 ± 0.33	60.80 ± 0.98	0.86 ± 0.00	<0.01
FDG	64	3.09 ± 0.42	71.39 ± 0.65	69.17 ± 1.14	73.60 ± 0.90	0.74 ± 0.01	87.74 ± 0.43	47.05 ± 0.90	0.76 ± 0.01	<0.01
DTI	64	98.15 ± 30.63	57.77 ± 1.10	60.43 ± 2.96	55.11 ± 2.12	0.59 ± 0.01	81.19 ± 0.88	31.08 ± 0.96	0.59 ± 0.01	0.14 ± 0.03

**Figure 5:**
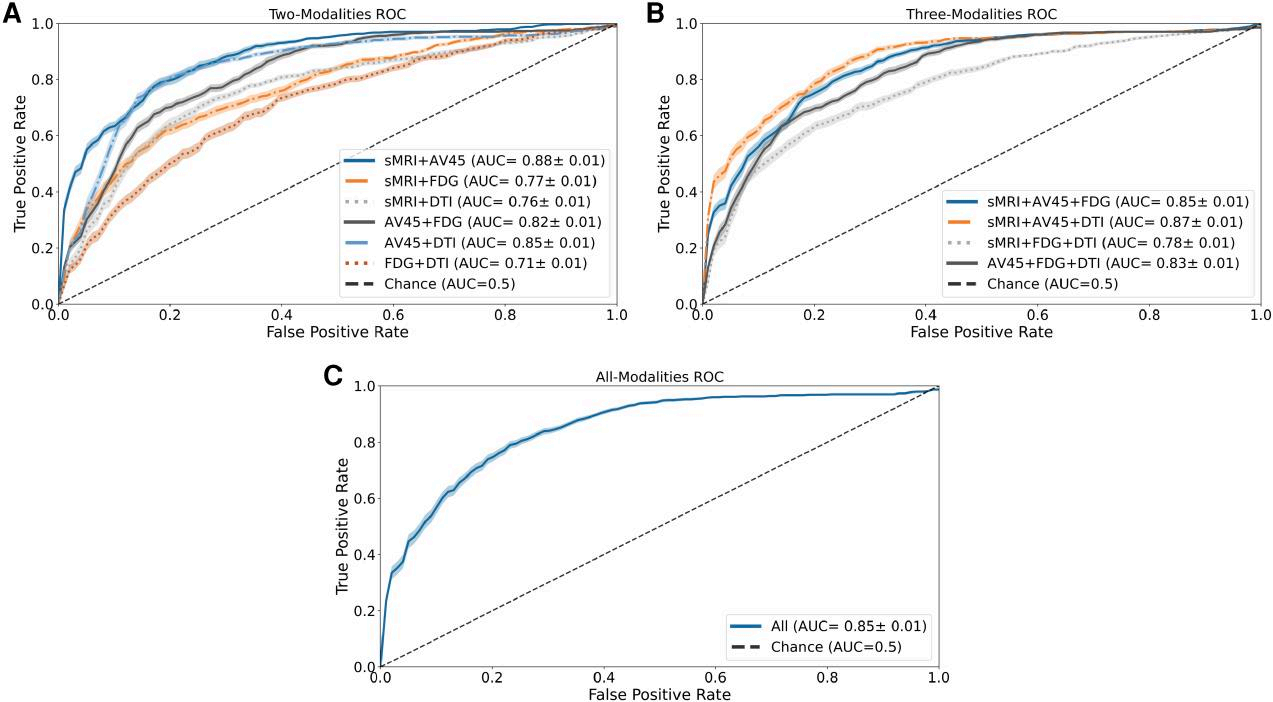
**Receiver operating characteristic (ROC) curve for all the multi-modality models developed using the models built using the most sensitive biomarkers.** This figure presents the ROC curve and the corresponding area under the curve (AUC) values for different combinations of the four neuroimaging modalities included in this study: structural magnetic resonance imaging (sMRI), fluorodeoxyglucose positron emission tomography (FDG-PET), florbetapir positron emission tomography (AV45-PET) and diffusion tensor imaging (DTI). The models were constructed using the identified most sensitive biomarkers for each modality. (**A**) displays the ROC curves for two-modality combinations; (**B**) shows the ROC curves for three-modality combinations; (**C**) exhibits the ROC curve for the combination of all four modalities. The ROC curves visualize the performance of each model while the AUC values provide a comprehensive metric for comparing the discriminative abilities of the different models.

Furthermore, we evaluated these new models in the previously mentioned different multi-modality combinations and summarized the results in Table 5. The respective ROC curves can be found in Fig. 5. Comparing these results with the previous ones, we observe that not a single combination benefited from the selection of the most sensitive biomarkers. Despite this, these new models show an overall better specificity value, with the exception of the combination of AV45-PET with FDG-PET, and improved PPV values, except for the combinations of FDG-PET with DTI and sMRI with FDG-PET, compared to the older models. However, this improvement comes at the cost of a generalized decrease in the Sensitivity and NPV values, which are lower across all models. Finally, analysing the new models’ ROC curves, it is possible to see a similar pattern to the case of using all features. Regarding the two-modality combinations, there seem to be two clusters of ROC curves. The first one comprises the combinations of sMRI with FDG-PET, sMRI with DTI and FDG-PET with DTI, closer to the chance line with AUC values ranging from 0.71 to 0.77. The second cluster comprises the remainder of the combinations further from the chance line, reaching AUC values in the range of 0.82 to 0.88.

**Table 5:** Performance of the different multi-modal models using the most sensitive biomarkers as well as with all features

Combination	Acc	Sen	Spe	F1-score	NPV	PPV	AUC	*P*-value
Sensitive biomarkers								
** sMRI + AV45**	66.76 ± 1.21	36.07 ± 2.81	97.45 ± 0.67	0.79 ± 0.01	81.96 ± 0.62	88.43 ± 2.43	0.88 ± 0.01	<0.01
** sMRI + FDG**	70.42 ± 0.89	60.30 ± 1.66	80.54 ± 0.72	0.76 ± 0.01	85.70 ± 0.55	51.48 ± 1.04	0.77 ± 0.01	<0.01
** sMRI + DTI**	58.39 ± 1.56	21.11 ± 3.75	95.67 ± 0.79	0.71 ± 0.01	78.43 ± 0.75	61.72 ± 5.04	0.76 ± 0.01	0.44 ± 0.20
** AV45 + FDG**	67.18 ± 0.74	43.45 ± 1.45	90.91 ± 0.39	0.77 ± 0.01	82.57 ± 0.40	61.93 ± 1.23	0.82 ± 0.01	<0.01
** AV45 + DTI**	51.66 ± 0.28	3.67 ± 0.63	99.65 ± 0.13	0.66 ± 0.00	75.25 ± 0.19	85.79 ± 5.79	0.85 ± 0.01	0.16 ± 0.10
** FDG + DTI**	59.62 ± 1.02	28.02 ± 2.38	91.23 ± 0.89	0.72 ± 0.01	78.96 ± 0.52	53.58 ± 2.70	0.71 ± 0.01	0.39 ± 0.15
** sMRI + AV45 + FDG**	70.05 ± 1.05	47.18 ± 2.23	92.93 ± 0.53	0.80 ± 0.01	83.92 ± 0.57	69.83 ± 1.49	0.85 ± 0.01	<0.01
** sMRI + AV45 + DTI**	57.59 ± 1.26	15.62 ± 2.60	99.56 ± 0.21	0.71 ± 0.01	77.73 ± 0.56	91.17 ± 1.49	0.87 ± 0.01	<0.01
** sMRI + FDG + DTI**	67.20 ± 1.10	42.01 ± 2.36	92.40 ± 0.56	0.78 ± 0.01	82.54 ± 0.58	65.29 ± 1.67	0.78 ± 0.01	0.02 ± 0.01
** AV45 + FDG + DTI**	57.76 ± 0.53	17.47 ± 1.11	98.06 ± 0.35	0.72 ± 0.01	77.75 ± 0.26	78.68 ± 3.34	0.83 ± 0.01	0.08 ± 0.03
** All**	62.72 ± 1.13	27.56 ± 2.45	97.88 ± 0.37	0.76 ± 0.01	80.01 ± 0.54	84.79 ± 2.42	0.85 ± 0.01	0.02 ± 0.01
All features								
** sMRI + AV45**	76.02 ± 0.77	65.35 ± 1.47	86.69 ± 0.74	0.81 ± 0.01	88.26 ± 0.44	62.88 ± 1.38	0.86 ± 0.01	<0.01
** sMRI + FDG**	78.43 ± 0.77	77.12 ± 1.07	79.75 ± 0.86	0.80 ± 0.01	91.23 ± 0.40	56.53 ± 1.24	0.83 ± 0.01	<0.01
** sMRI + DTI**	71.42 ± 0.75	58.11 ± 1.41	84.74 ± 0.76	0.78 ± 0.01	85.86 ± 0.40	56.62 ± 1.26	0.77 ± 0.01	<0.01
** AV45 + FDG**	74.67 ± 0.71	62.71 ± 1.22	86.63 ± 0.97	0.81 ± 0.01	87.44 ± 0.38	62.54 ± 1.91	0.85 ± 0.01	<0.01
** AV45 + DTI**	52.27 ± 0.42	6.99 ± 1.00	97.55 ± 0.32	0.67 ± 0.01	75.84 ± 0.29	47.24 ± 5.29	0.85 ± 0.00	1.02 ± 0.25
** FDG + DTI**	71.12 ± 0.62	55.79 ± 1.30	86.46 ± 0.81	0.79 ± 0.01	85.44 ± 0.37	58.78 ± 1.30	0.75 ± 0.01	<0.01
** sMRI + AV45 + FDG**	78.79 ± 0.84	68.46 ± 1.31	89.12 ± 0.69	0.84 ± 0.01	89.40 ± 0.46	68.41 ± 1.52	0.88 ± 0.01	<0.01
** sMRI + AV45 + DTI**	73.24 ± 0.70	48.88 ± 1.43	97.61 ± 0.49	0.84 ± 0.01	85.14 ± 0.36	88.85 ± 1.91	0.86 ± 0.01	<0.01
** sMRI + FDG + DTI**	72.53 ± 0.92	57.33 ± 1.48	87.73 ± 0.77	0.80 ± 0.01	86.00 ± 0.48	61.70 ± 1.79	0.82 ± 0.01	<0.01
** AV45 + FDG + DTI**	69.29 ± 0.64	43.70 ± 1.35	94.88 ± 0.58	0.80 ± 0.00	83.48 ± 0.36	76.01 ± 2.17	0.84 ± 0.01	<0.01
** All**	71.94 ± 0.77	50.57 ± 1.50	93.31 ± 0.54	0.81 ± 0.01	85.00 ± 0.41	72.54 ± 1.79	0.87 ± 0.01	<0.01

The combinations that benefited from the usage of the sensitive biomarkers are highlighted in bold. Acc, balance accuracy; Sen, sensitivity; Spe, specificity; NPV, negative predictive value; PPV, positive predictive value; AUC, area under the curve.

## Discussion

### Single-modality analysis

In this project, our focus was on studying the predictive ability of different imaging biomarkers derived from four imaging modalities: MRI, AV45-PET, FDG-PET and DTI, in relation to the conversion from MCI to Alzheimer’s disease. Previous studies have primarily examined single-modality biomarkers for predicting this conversion.^16,35,49-54^

Based on the prevailing theoretical model, inspired by the ADNI initiative, which explains the temporal ordering of Alzheimer’s disease biomarkers,^55^ both amyloid deposition and decreased glucose metabolism are considered biomarkers that show alterations before the onset of symptoms, while volumetric changes tend to occur much later. Our results demonstrate that AV45-PET, FDG-PET and sMRI can be used to extract features associated with these biomarkers and predict the conversion from MCI to Alzheimer’s disease (see Table 2). Furthermore, we evaluated the performance of DTI imaging, which has recently shown promising complementary potential by providing independent and joint information for both Alzheimer’s disease classification^15^ and in the prediction of progression.^30^

Comparing the individual performances of the best models from each imaging modality, we observed that the AV45-PET model exhibited the highest predictive performance, followed by the sMRI, FDG-PET and DTI models. These results align with the temporal notion that amyloid deposition, glucose hypometabolism and volumetric changes during the prodromal stages of Alzheimer’s disease can be captured through non-invasive imaging and used for predicting the conversion to Alzheimer’s disease. Surprisingly, the best sMRI-based models exhibited similar performance to FDG-PET. This may be partly due to the larger amount of data available for sMRI modality. Additionally, these findings support the theoretical model regarding the temporal ordering of changes in Alzheimer’s disease biomarkers.

When analysing our single-modality models, it is important to note that all the DTI-based models were unable to consistently perform above chance (*P* < 0.05), with the best model (MD-RBF) only managing a mean *P*-value of 0.14 and only half of the models performing above chance. We assume that this low performance is partly due to the lack of data, as DTI is the modality with fewer samples. Despite this, we included this model in further analysis to try and extrapolate if this modality could, in fact, provide complementary information to the classification problem. However, further studies should be carried out concerning DTI data and feature extraction methods to identify the optimal approaches that can consistently perform above the chance level.

Regarding the other modalities, the AV45-PET-based models clearly outperform the FDG-PET- and sMRI-based models, reaching a mean balanced accuracy value as high as 83.51%. Furthermore, these models show a remarkable ability to successfully predict if a participant will not convert to Alzheimer’s disease, reaching NPV values as high as 95.10%. This ability seems to be intrinsic to the other imaging modalities as well since both the best-performing models from sMRI and FDG-PET also reach mean NPV values in the high 80s%, with the best models reaching 87.67% and 88.74%, respectively. However, all imaging modalities alone are unable to predict with considerable certainty which individuals will convert to Alzheimer’s disease.

### Multi-modality analysis

In addition to evaluating single-modality models, we also explored the effects of combining different imaging modalities in predicting the conversion from MCI to Alzheimer’s disease. To achieve this, we constructed and trained one new model for each imaging modality using the feature set that resulted in the best overall performance. These four models, one for each imaging modality, were then combined using a simple weighted ensemble technique. We set equal weights for all classifiers, assuming that each classifier had the same importance for the final prediction and that the information from different imaging sources had the same impact on the final classification. In this framework, each classifier acted as an expert, providing an opinion based solely on the information from its respective imaging data, which allowed us to directly compare the performance of distinct imaging modalities and their combination in predicting the conversion from MCI to Alzheimer’s disease.

Multi-modality studies can be challenging to conduct, especially when considering only imaging biomarkers, as obtaining data from all modalities for the same patients can be difficult due to various reasons, such as equipment availability, cost, lack of patient consent and patient dropouts in longitudinal studies. However, some studies have managed to conduct multi-modal studies, mainly using sMRI data combined with other imaging modalities or other physiological biomarkers.^19,20,31-35,56^

Our results showed that not all multi-modal combinations led to an improvement in predicting which MCI subjects will convert to Alzheimer’s disease (see Table 3). Among the two-modality combinations, surprisingly the combination of sMRI with AV45-PET did not result in a better predictive performance compared to the base classifiers, contrary to data from other studies where this combination improved the overall performance of the model.^35^ Possible justifications for this result could be the discrepancy in the performance of the two base models and the fusion strategy used to combine them. Equal weighting of each imaging modality may not be optimal as some modalities may better capture the underlying pathophysiology of Alzheimer’s disease. This lack of improvement was also observed in all other combinations except for sMRI with FDG-PET, which showed an overall improvement in classification ability. This finding might suggest that the best combination of neuroimaging biomarkers (sMRI with AV45-PET) for predicting the diagnosis of AD is not necessarily the same to predict conversion. However, it’s worth noting that AV45-PET alone still exhibited better performance than the combination of sMRI with FDG-PET. Nevertheless, all combinations showed an improvement in specificity and, more importantly, in PPV. This suggests that combining different imaging modalities might help identify which MCI individuals will indeed convert to Alzheimer’s disease. Additionally, we observed an improvement in the F1-score for all combinations where AV45-PET was not present, indicating that data from different imaging modalities may introduce complementary information for better identification of which MCI individuals will convert to Alzheimer’s disease.

Another interesting finding from our study is related to the performance of the DTI-based classifier when combined with other imaging modalities. While the DTI model demonstrated a significant performance discrepancy when compared to the sMRI and FDG models individually, its combination with these modalities led to an improvement in both specificity and PPV. This result suggests that the combination of DTI with sMRI or FDG-PET provided complementary information, enhancing the identification of MCI individuals who will convert to Alzheimer’s disease. However, the situation was markedly different when DTI was combined with AV45-PET. In this case, we observed a decrease in the overall classification performance of the model, with a particularly alarming consequence—the model’s complete inability to correctly classify any participant who will convert from MCI to Alzheimer’s disease (sensitivity = 6.99%). This finding is consistent with our previous results, which indicated that the combination of DTI and amyloid PET does not provide complementary information, suggesting that both modalities convey non-independent information regarding the prediction of MCI to Alzheimer’s disease conversion and the classification of Alzheimer’s disease.^15^ On a positive note, we observed that the combination of sMRI with FDG-PET yielded an improvement in the prediction capabilities of the combined model that is also reported in other studies.^35^ This outcome further validates the potential benefits of combining specific imaging modalities to enhance prediction accuracy for Alzheimer’s disease conversion. It is also important to acknowledge the challenges in directly comparing our results with those of other studies due to the unique nature of our research design and fusion strategy. Since we evaluated all possible combinations of the four imaging modalities, including data from each one in the fusion approach, we are not aware of any previous studies that have directly compared the performance and combinations of all these modalities. This highlights the novelty and significance of this work, as it provides comprehensive insights into the potential benefits and limitations of multi-modal approaches for predicting the conversion from MCI to Alzheimer’s disease.

The results of combining three modalities showed outcomes similar to the two-modality approach, with no combinations outperforming the best single-modality model. Due to the novelty of our work, there is a lack of studies for direct comparison. Nonetheless, we explored the impact of adding one more modality to the previously explored combinations. As observed in the two-modality results, the combination of three modalities improved both the specificity and PPV of the final classifier compared to the base classifiers and the previous two-modality models. This reinforces the idea that combining different imaging modalities might be beneficial in identifying which MCI individuals will convert to Alzheimer’s disease. We also found an improvement in the F1-score for all combinations except for the combination of AV45-PET with FDG-PET and DTI. Notably, the combination of sMRI with AV45-PET and DTI resulted in the model with the highest PPV, reaching a mean value of 88.85%. Furthermore, this combination also showed robust mean NPV (85.14%) and specificity (97.61%) values. However, this came at the cost of a lower sensitivity mean value of 48.88%. This combination model is interesting because, although it cannot successfully identify all individuals who will convert to Alzheimer’s disease, when it does identify an individual who will convert, it has a great probability of being correct. Additionally, the combination of sMRI with both PET modalities (AV45 and FDG) outperformed all the two-modality combinations that can be achieved using these modalities. It achieved a balanced accuracy of 78.79%, with a mean PPV value of 68.41% and a mean specificity value of 89.12%, higher than any of the single-modality models. Overall, there was a general improvement in all classification metrics at the cost of a decrease in sensitivity.

Regarding the combination of all four imaging modalities, it followed a similar trend, achieving a mean balanced accuracy value of 71.94%, which is lower than the single models of sMRI (72.75%) and AV45-PET (83.51%) and lower than most of the previous combinations, except for a few specific three-modality combinations. However, it showed interesting specificity (93.31%), NPV (85.00%) and PPV (72.54%) mean values, similar to those found in the three-modality combinations. This suggests that blindly adding more information will not necessarily improve the overall prediction capabilities. Fine-tuning the weights of each modality may be necessary to optimize the model’s performance for specific classification metrics, depending on the model’s intended use.

Currently, our models are good at identifying all the MCI individuals who will not convert, but it would be more valuable if the models could accurately identify which MCI individuals will indeed convert to Alzheimer’s disease. Adjusting the decision threshold may help achieve better models, tailored to maximize specific classification metrics based on the model’s intended application. This, in conjunction with the previous findings, suggests that carefully fine-tuning the weights given to all modalities and the decision threshold could potentially improve the performance of all previous combinations to reach their true potential.

Furthermore, the ROC curve analysis of all the models (Figs 1 and 2) also indicates that adjusting the decision threshold could help achieve better models tailored to maximize specific classification metrics, depending on the model’s future utility. These results, along with the previous findings, reinforce the idea that it might be possible to improve all previous combinations by carefully fine-tuning the weights given to all modalities and the decision threshold. However, for the purpose of this work, the focus was on evaluating the effects of equally combining the different imaging modalities, providing valuable insights into the potential benefits and limitations of multi-modal approaches for predicting the conversion from MCI to Alzheimer’s disease.

### Classification using the subset of the most sensitive biomarkers

In our investigation, we went beyond evaluating the performance of various imaging modalities and their combinations and delved into a more in-depth analysis of the most sensitive biomarkers derived from the pool of features available. This exploration was aimed at identifying the specific regions that hold significant potential for predicting the conversion from MCI to Alzheimer’s disease, providing valuable insights into the underlying disease pathology. To achieve this, we employed a ranking approach for each feature across all the imaging modalities, resulting in a comprehensive evaluation of the most sensitive biomarkers. As we examined the results (Supplementary Table 5), we observed that the top-ranking features for all imaging modalities corresponded to regions that have well-established biological relevance in the context of Alzheimer’s disease.

Zooming in on the most sensitive biomarkers derived from sMRI, we encountered regions that held immense promise for early Alzheimer’s disease detection. At the pinnacle of the list, we found the amygdala and the hippocampus, bilaterally. These subcortical regions have been extensively studied in the context of Alzheimer’s disease pathology and have been consistently associated with the disease’s progression.^57,58^ Moreover, their deterioration has been observed in the early stages of Alzheimer’s, underscoring their significance as potential early indicators of the disease. The inclusion of these regions as sensitive biomarkers in our study further reinforced their clinical relevance and provided valuable confirmation of their role in predicting the conversion from MCI to Alzheimer’s disease. Furthermore, our analysis also revealed the appearance of the right posterior temporal lobe as a sensitive biomarker, which garnered significant interest due to recent findings of reduced functional connectivity in this region among individuals with Alzheimer’s disease.^59^ Our study adds novel evidence, suggesting that structural changes in the temporal lobe could serve as early signs of Alzheimer’s disease and may prove instrumental in predicting the conversion of MCI individuals to the disease. Another funding in our evaluation was the identification of the brainstem as a sensitive biomarker. Existing literature provided compelling evidence linking the dorsal raphe nucleus (DRN), located in the midbrain and pons, with early Alzheimer’s disease.^60,61^ The DRN contains a substantial portion of brainstem serotonergic neurons, known to be dysfunctional in Alzheimer’s disease, further solidifying its potential as a promising biomarker for early disease detection or prediction of MCI conversion to Alzheimer’s disease.^62,63^ The inclusion of the brainstem as a potential biomarker in our study emphasizes the importance of examining regions beyond the traditional cortical regions in the context of Alzheimer’s disease progression.

Regarding the AV45-PET most sensitive biomarkers, we identified the presence of subcortical regions, particularly the putamen and nucleus accumbens, at the top of the list. These findings shed light on the early events in Alzheimer’s disease pathogenesis, where the deposition of amyloid plaques has been recognized as a hallmark.^64^ The appearance of the putamen structure as a sensitive biomarker came as no surprise, given the extensive research on the alterations observed in this subcortical region in the context of Alzheimer’s disease. Specifically, studies have consistently demonstrated a significant reduction in the volume of the putamen in individuals diagnosed with probable Alzheimer’s disease, providing strong evidence of its involvement in the disease’s progression. Moreover, the specific accumulation of β-amyloid in the putamen during the pathophysiological development of Alzheimer’s disease, but not during normal aging, has reinforced its potential as a sensitive biomarker for early detection and prediction of Alzheimer’s disease.^65^ As to the nucleus accumbens, our analysis revealed its prominent role as a sensitive biomarker in AV45-PET imaging. Recent studies have unveiled intriguing findings regarding volumetric and shape atrophy in the nucleus accumbens among individuals diagnosed with MCI and Alzheimer’s disease.^66^ The presence of these alterations in this structure, which is part of the brain’s reward system, suggests potential disruptions to its normal function in the early stages of Alzheimer’s disease. Further supporting this notion, studies in rats have shown that artificially increasing β-amyloid levels in the brain resulted in increased phasic dopamine release in the shell of the nucleus accumbens.^67^ This compelling evidence further implicates the nucleus accumbens in the pathogenesis of Alzheimer’s disease and highlights its potential as a sensitive biomarker for early disease detection and prediction of MCI conversion to Alzheimer’s disease.

Considering the FDG-PET most sensitive biomarkers, we observed both familiar and novel regions that hold significance in the context of Alzheimer’s disease. Once again, the amygdala and the hippocampus emerge as crucial regions at the top of the list, reaffirming their well-established role in Alzheimer’s disease pathology. These subcortical structures have consistently been identified as key regions that are severely and consistently affected in Alzheimer’s disease pathology, with their deterioration being detected even in the early stages of the disease.^57,58^ As such, their prominence as sensitive biomarkers in FDG-PET imaging adds clinical meaning to our findings and underscores their potential utility in predicting the conversion from MCI to Alzheimer’s disease.

A new addition to the list of FDG-PET sensitive biomarkers is the posterior cingulate gyrus, where we can find the posterior cingulate cortex (PCC). The PCC is an important region situated in the posterior part of the cingulate gyrus, exhibiting higher metabolic activity and dense anatomical and functional connections to many other regions in the brain. Recent studies have shed light on the involvement of the PCC in Alzheimer’s disease pathogenesis, reporting PCC hypometabolism, volume atrophy and connectivity corruption in patients with AD.^68^ Notably, investigations using MRI imaging have consistently identified the PCC as one of the most vulnerable regions in the early stages of Alzheimer’s disease.^69^ Our results align with these findings, further substantiating the notion that PCC hypometabolism may serve as a reliable indicator of early stages of Alzheimer’s disease pathogenesis and could aid in predicting which MCI individuals will eventually convert to Alzheimer’s disease. Furthermore, our study highlights the reappearance of the brainstem as a sensitive biomarker in FDG-PET imaging, as observed previously when analysing the sMRI sensitive biomarkers. The brainstem’s significance in Alzheimer’s disease pathophysiology is underscored by the reduction in dorsal midbrain volume observed in Alzheimer’s disease patients, which corresponds to the location of the raphe nuclei.^70^ Notably, while there is no previous evidence suggesting alterations in brainstem metabolism in Alzheimer’s disease patients, it is important to consider that, like other regions affected by Alzheimer’s disease, the loss of neuronal mass in the brainstem will inevitably lead to reduced overall glucose uptake in the affected region.

Finally, in the realm of DTI imaging, we delved into a novel set of brain regions that emerged as highly sensitive biomarkers for predicting the conversion from MCI to Alzheimer’s disease. Remarkably, the corpus callosum surfaced as the most sensitive DTI biomarker, warranting particular attention in understanding its potential role in the early detection of Alzheimer’s disease. The corpus callosum is a vital structure responsible for facilitating communication and information transfer between the left and right cerebral hemispheres. Its importance in interhemispheric connectivity and integration of cognitive processes has long been recognized. In light of this, previous studies have found morphological and shape changes of the corpus callosum in the context of Alzheimer’s disease progression.^71^ Building upon these prior findings, microstructural analysis conducted using DTI has unveiled alterations in diffusion patterns within the region of the corpus callosum, which may be indicative of demyelination and axonal degeneration in patients with Alzheimer’s disease.^72^ Our results build upon these previous findings suggesting that microstructural changes in the corpus callosum can be used as a potential biomarker for predicting the conversion of MCI to Alzheimer’s disease. Further future exploration into the mechanisms underlying the corpus callosum’s involvement in Alzheimer’s disease pathogenesis may offer valuable insights into disease progression and contribute to the development of more accurate diagnostic and prognostic tools. Alongside the corpus callosum, the postcentral gyrus emerged as another intriguing sensitive biomarker in DTI imaging. The postcentral gyrus, located in the parietal lobe, is renowned for its role in processing sensory information and integrating somatosensory signals. In the context of Alzheimer’s disease, little information is currently available regarding its specific connection to MCI or Alzheimer’s disease. However, a recent study reported functional connectivity differences in the postcentral gyrus between normal controls and participants with MCI and Alzheimer’s disease.^73^ This finding is particularly intriguing when considering the application of DTI as a biomarker. Demyelination and axonal degeneration, as seen in Alzheimer’s disease, may hinder efficient communication between different brain regions, leading to a decrease in functional connectivity. The identification of the postcentral gyrus as a sensitive biomarker may open up new avenues for exploring the intricate network disruptions and neurodegenerative processes that characterize Alzheimer’s disease.

In this final phase of our analysis, we took a closer look at the potential of sensitive biomarkers by developing new models using the optimal number of these biomarkers, as determined by the BIC criteria. The goal was to investigate whether these selected biomarkers could significantly enhance the classification performance of our models. We found that simply focusing on the sensitive biomarkers were enough to achieve accuracies close to the original models built using all available features. This finding implies that the selected sensitive biomarkers carry a wealth of information crucial for the model’s decision-making process. By concentrating on these key biomarkers, we can still achieve comparable performance to the more comprehensive models, indicating the significance of these specific biomarkers in capturing the essential patterns related to MCI to Alzheimer’s disease conversion. Continuing with our investigation, we proceeded to explore the effects of combining the new models using the same multi-modal approaches previously applied to the old models. As seen in Table 5, the combined models did not exhibit an overall improvement in their classification abilities. However, our analysis unveiled an interesting trend in the performance metrics. While the overall classification accuracy did not significantly change, we observed notable enhancements in both sensitivity and PPV for the new combinations. These results suggest that the sensitive biomarkers play a pivotal role in capturing the underlying processes that facilitate the accurate prediction of which MCI individuals will convert to Alzheimer’s disease. Although the overall performance of the multi-modal combinations did not see a drastic improvement, the focus on sensitive biomarkers appeared to fine-tune the models, enabling them to better identify true positive cases (higher sensitivity) and make more accurate positive predictions (higher PPV). This discovery highlights the potential value of sensitive biomarkers in refining predictive models for Alzheimer’s disease conversion. In the future, it will be essential to delve deeper into the specific biological significance of these sensitive biomarkers and their relationships with the underlying pathophysiological processes of Alzheimer’s disease. Moreover, future investigations could explore alternative fusion strategies, such as optimizing the weights assigned to different modalities, to unlock the full potential of multi-modal approaches in predicting Alzheimer’s disease conversion. Exploring these approaches should lead to more precise and personalized diagnostic tools, providing early detection and intervention opportunities for individuals at risk of developing Alzheimer’s disease.

## Conclusion

In this study, we introduce a pioneering framework that systematically evaluates and compares different imaging modalities to identify individuals with MCI likely to progress to Alzheimer’s disease. Our analysis highlights AV45-PET, measuring β-amyloid burden, as a promising neuroimaging biomarker for conversion and early Alzheimer’s detection. The study emphasizes the value of combining diverse modalities, revealing both complementary information and potential redundancies. For instance, while DTI individually showed weaker performance, its combination with sMRI and FDG-PET significantly improved specificity and PPV, suggesting unique contributions.

Additionally, our findings on relevant biomarkers provide intriguing insights into Alzheimer’s early pathophysiology. The brainstem’s presence in both sMRI and FDG-PET as a sensitive biomarker suggests Alzheimer’s origins in the middle brainstem. The unexpected significance of the corpus callosum and adjacent cortical regions in DTI merits further exploration, offering potential new perspectives on Alzheimer’s structural changes. In conclusion, our multi-modal approach offers a comprehensive assessment, highlighting AV45-PET’s promise, the benefits of combining modalities and the importance of integrating sensitive biomarkers for enhanced predictive models.

This study significantly contributes to neuroimaging and Alzheimer’s research, paving the way for more accurate and personalized diagnostic tools for early Alzheimer’s detection and management.

## Supplementary Material

fcae208_Supplementary_Data

## Data Availability

The ADNI data used in this study were obtained from the ADNI database (adni.loni.usc.edu). All the data used in this study are available from the corresponding author, upon reasonable request.
