# Supplementary material for: Predicting conversion from mild cognitive impairment to Alzheimer’s disease: a multimodal approach
Source: Brain Commun. 2024 Jun 14;6(4):fcae208. doi: 10.1093/braincomms/fcae208 (PMC11220508; doi:10.1093/braincomms/fcae208)
Supplement: fcae208_Supplementary_Data [file fcae208_supplementary_data.docx]

**Supplementary material:**

**Predicting Conversion from Mild Cognitive Impairment to Alzheimer’s Disease: a multimodal approach**

**Supplementary Figure 1. Schematic of participant inclusion criteria and labelling.** This figure illustrates the process of including mild cognitive impairment (MCI) (n=486) participants in the study and classifying them as stable MCI (sMCI) (n=252) or converted MCI (cMCI) (n=234).

**
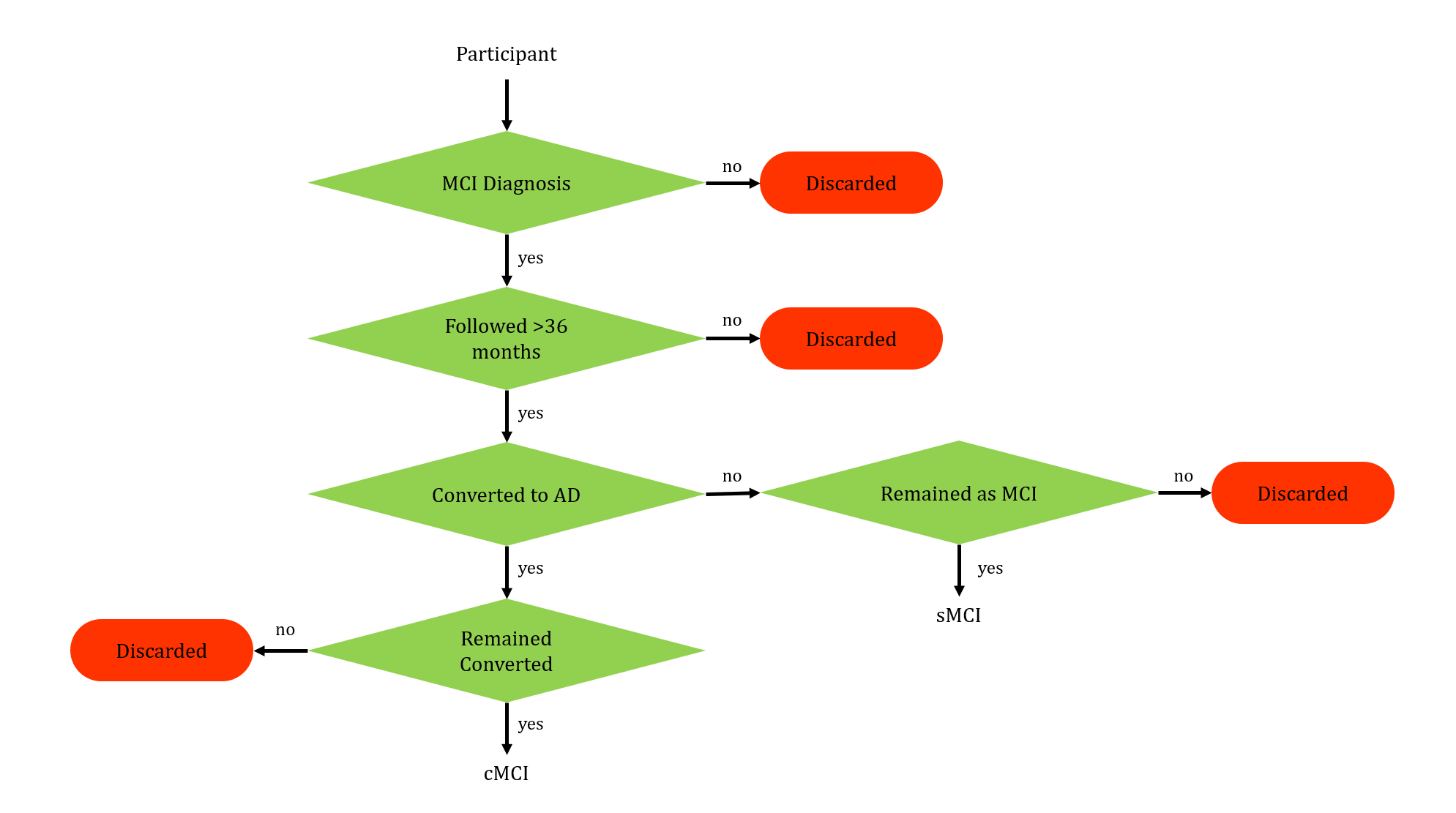
**

| 002_S_0729 | 013_S_4395 | 023_S_4122 | 035_S_0997 | 057_S_1007 | 094_S_0921 | 123_S_4780 | 130_S_2373 |
| --- | --- | --- | --- | --- | --- | --- | --- |
| 002_S_0782 | 013_S_4595 | 023_S_4502 | 035_S_2061 | 057_S_1217 | 094_S_1314 | 123_S_4806 | 130_S_2391 |
| 002_S_1070 | 014_S_0169 | 023_S_4796 | 035_S_2074 | 057_S_1265 | 094_S_1417 | 126_S_0708 | 130_S_2403 |
| 002_S_1155 | 014_S_0557 | 024_S_1393 | 035_S_4582 | 057_S_1269 | 094_S_2216 | 126_S_0709 | 130_S_4250 |
| 002_S_1268 | 014_S_0563 | 024_S_2239 | 035_S_4784 | 057_S_2398 | 094_S_2238 | 126_S_0865 | 130_S_4294 |
| 002_S_4229 | 014_S_0658 | 024_S_4169 | 036_S_0656 | 057_S_4888 | 094_S_4162 | 126_S_1077 | 130_S_4415 |
| 002_S_4447 | 014_S_2185 | 024_S_4674 | 036_S_0673 | 062_S_1182 | 094_S_4630 | 126_S_1187 | 130_S_4417 |
| 002_S_4473 | 014_S_2308 | 027_S_0116 | 036_S_0869 | 062_S_1299 | 094_S_4858 | 126_S_2360 | 130_S_4817 |
| 002_S_4521 | 014_S_4263 | 027_S_0179 | 036_S_0945 | 067_S_0077 | 098_S_0160 | 126_S_2405 | 131_S_1389 |
| 002_S_4654 | 016_S_0769 | 027_S_0256 | 036_S_0976 | 067_S_0098 | 098_S_0269 | 126_S_2407 | 133_S_0638 |
| 003_S_0908 | 016_S_1117 | 027_S_0307 | 036_S_2380 | 067_S_0176 | 098_S_0667 | 126_S_4458 | 133_S_0727 |
| 003_S_1057 | 016_S_1138 | 027_S_0408 | 036_S_4538 | 067_S_0290 | 098_S_2047 | 126_S_4507 | 133_S_0771 |
| 003_S_1122 | 016_S_1326 | 027_S_0644 | 036_S_4714 | 067_S_0336 | 098_S_2052 | 126_S_4514 | 133_S_0792 |
| 003_S_2374 | 016_S_2031 | 027_S_0835 | 036_S_4715 | 067_S_0607 | 098_S_2079 | 126_S_4675 | 133_S_0912 |
| 003_S_4354 | 016_S_4902 | 027_S_1045 | 036_S_4736 | 067_S_2196 | 099_S_0051 | 126_S_4712 | 135_S_4281 |
| 005_S_0324 | 016_S_5031 | 027_S_1387 | 037_S_0150 | 067_S_4782 | 099_S_0054 | 126_S_4891 | 135_S_4309 |
| 005_S_0448 | 018_S_0057 | 027_S_2183 | 037_S_0377 | 068_S_0478 | 099_S_0291 | 126_S_4896 | 135_S_4356 |
| 005_S_0572 | 018_S_0080 | 027_S_2219 | 037_S_0501 | 068_S_0802 | 099_S_1034 | 127_S_0394 | 135_S_4406 |
| 005_S_1224 | 018_S_0142 | 027_S_2245 | 037_S_0539 | 068_S_2168 | 099_S_2063 | 127_S_0925 | 135_S_4489 |
| 005_S_2390 | 018_S_0155 | 027_S_2336 | 037_S_0588 | 068_S_4067 | 099_S_4157 | 127_S_1032 | 135_S_4689 |
| 005_S_4185 | 018_S_0406 | 027_S_4729 | 037_S_1078 | 068_S_4431 | 099_S_4202 | 127_S_1140 | 135_S_4722 |
| 006_S_0675 | 018_S_2133 | 027_S_4804 | 037_S_1421 | 072_S_1211 | 099_S_4205 | 127_S_1419 | 135_S_4723 |
| 006_S_1130 | 018_S_2155 | 027_S_4869 | 037_S_4015 | 072_S_1380 | 099_S_4463 | 127_S_1427 | 136_S_0107 |
| 006_S_4346 | 018_S_4809 | 027_S_4873 | 037_S_4030 | 072_S_2037 | 099_S_4498 | 127_S_2213 | 136_S_0195 |
| 006_S_4363 | 018_S_4868 | 027_S_4919 | 037_S_4146 | 072_S_2072 | 100_S_0006 | 127_S_4197 | 136_S_0695 |
| 006_S_4679 | 018_S_4889 | 027_S_4926 | 037_S_4432 | 072_S_2083 | 100_S_0296 | 127_S_4210 | 136_S_0873 |
| 007_S_0041 | 019_S_4548 | 027_S_4936 | 041_S_0314 | 072_S_2116 | 100_S_0892 | 127_S_4240 | 136_S_0874 |
| 007_S_0101 | 021_S_0141 | 027_S_4966 | 041_S_1010 | 072_S_2164 | 100_S_4556 | 127_S_4301 | 136_S_1227 |
| 007_S_0128 | 021_S_0231 | 029_S_0878 | 041_S_1260 | 072_S_4063 | 109_S_0950 | 127_S_4765 | 137_S_0158 |
| 007_S_0249 | 021_S_0273 | 029_S_0914 | 041_S_1418 | 072_S_4206 | 109_S_1114 | 127_S_4844 | 137_S_0481 |
| 007_S_0293 | 021_S_0276 | 029_S_1073 | 041_S_1425 | 072_S_4226 | 109_S_1183 | 127_S_4928 | 137_S_0631 |
| 007_S_0344 | 021_S_0424 | 029_S_1218 | 041_S_4143 | 072_S_4383 | 109_S_2200 | 128_S_0200 | 137_S_0800 |
| 007_S_0698 | 021_S_0626 | 029_S_1318 | 041_S_4271 | 072_S_4390 | 109_S_4455 | 128_S_0225 | 137_S_0973 |
| 007_S_2106 | 021_S_2077 | 029_S_1384 | 041_S_4510 | 072_S_4394 | 109_S_4531 | 128_S_0227 | 137_S_0994 |
| 007_S_2394 | 021_S_2100 | 031_S_0294 | 041_S_4720 | 072_S_4462 | 109_S_4594 | 128_S_0608 | 137_S_1414 |
| 007_S_4272 | 021_S_2124 | 031_S_0351 | 041_S_4876 | 072_S_4465 | 114_S_0378 | 128_S_0611 | 137_S_4299 |
| 007_S_4467 | 021_S_2125 | 031_S_0830 | 041_S_4974 | 072_S_4522 | 114_S_1106 | 128_S_0770 | 137_S_4331 |
| 007_S_4611 | 021_S_4245 | 031_S_0867 | 041_S_4989 | 072_S_4539 | 114_S_1118 | 128_S_1043 | 137_S_4536 |
| 009_S_1030 | 021_S_4402 | 031_S_1066 | 051_S_1072 | 072_S_4613 | 114_S_2392 | 128_S_1088 | 137_S_4596 |
| 009_S_2208 | 021_S_4659 | 031_S_4029 | 051_S_1131 | 072_S_4871 | 114_S_4404 | 128_S_1406 | 137_S_4623 |
| 009_S_2381 | 021_S_4744 | 031_S_4149 | 052_S_0671 | 072_S_4941 | 114_S_5047 | 128_S_1407 | 137_S_4631 |
| 009_S_4324 | 021_S_4857 | 031_S_4476 | 052_S_0952 | 073_S_0518 | 116_S_0361 | 128_S_2045 | 137_S_4678 |
| 009_S_5000 | 022_S_0004 | 031_S_4590 | 052_S_0989 | 073_S_0746 | 116_S_0649 | 128_S_2220 | 137_S_4815 |
| 010_S_0161 | 022_S_0961 | 031_S_4721 | 052_S_1346 | 073_S_0909 | 116_S_0752 | 128_S_4553 | 137_S_4816 |
| 011_S_0241 | 022_S_1394 | 032_S_0214 | 052_S_1352 | 073_S_2153 | 116_S_0834 | 128_S_4571 | 141_S_0697 |
| 011_S_0326 | 022_S_2167 | 032_S_0718 | 052_S_2249 | 073_S_2191 | 116_S_1243 | 128_S_4636 | 141_S_0851 |
| 011_S_0362 | 022_S_5004 | 032_S_0978 | 052_S_4626 | 073_S_2225 | 116_S_1271 | 128_S_4653 | 141_S_1004 |
| 011_S_0861 | 023_S_0030 | 032_S_2119 | 052_S_4807 | 073_S_2264 | 116_S_1315 | 128_S_4742 | 141_S_1052 |
| 011_S_1080 | 023_S_0042 | 032_S_4823 | 052_S_4885 | 073_S_4216 | 116_S_4167 | 128_S_5066 | 141_S_1255 |
| 011_S_1282 | 023_S_0126 | 033_S_0513 | 052_S_4944 | 073_S_4300 | 116_S_4175 | 129_S_1246 | 141_S_1378 |
| 011_S_4235 | 023_S_0217 | 033_S_0514 | 052_S_4945 | 073_S_4311 | 116_S_4635 | 129_S_2347 | 141_S_2333 |
| 011_S_4547 | 023_S_0331 | 033_S_0567 | 053_S_0389 | 073_S_4312 | 116_S_4898 | 129_S_4073 | 141_S_4160 |
| 011_S_4893 | 023_S_0376 | 033_S_0723 | 053_S_0507 | 073_S_4360 | 123_S_0050 | 129_S_4220 | 153_S_2109 |
| 012_S_0634 | 023_S_0388 | 033_S_0906 | 053_S_0621 | 073_S_4443 | 123_S_0108 | 129_S_4287 | 153_S_2148 |
| 012_S_4012 | 023_S_0604 | 033_S_0922 | 053_S_0919 | 073_S_4614 | 123_S_0390 | 130_S_0102 | 153_S_4621 |
| 012_S_4094 | 023_S_0625 | 033_S_1116 | 053_S_2396 | 073_S_4777 | 123_S_1300 | 130_S_0285 | 153_S_4838 |
| 012_S_4128 | 023_S_0887 | 033_S_1284 | 053_S_4557 | 073_S_4986 | 123_S_2055 | 130_S_0289 | 941_S_1295 |
| 012_S_4188 | 023_S_1046 | 035_S_0033 | 053_S_4661 | 082_S_2121 | 123_S_2363 | 130_S_0449 | 941_S_2060 |
| 012_S_4849 | 023_S_4035 | 035_S_0204 | 057_S_0464 | 082_S_2307 | 123_S_4127 | 130_S_0505 | 941_S_4036 |
| 012_S_4987 | 023_S_4115 | 035_S_0292 | 057_S_0839 | 094_S_0434 | 123_S_4170 | 130_S_0783 | 941_S_4764 |

**Supplementary Table 1: List of IDs of each participant in the ADNI dataset**

List of all the site IDs for all the participants used in this study.

**Supplementary Figure 2. Strategy used for splitting the included participants into the Incomplete Visits Set (IVS) and Complete Visits Set (CVS).** The stable mild cognitive impaired (sMCI) (n=252) and converted mild cognitive impaired (cMCI) (n=234) participants were divided into the Incomplete Visits Set (IVS) and Complete Visits Set (CVS), ensuring that each data set contained participants form both sMCI and cMCI. For admissibility to the CVS participants must have at least one session with data for structural magnetic resonance imaging (sMRI), Fluorodeoxyglucose (FDG) positron emission tomography (PET), Florbetapir (AV45-PET), and diffusion tensor imaging (DTI).


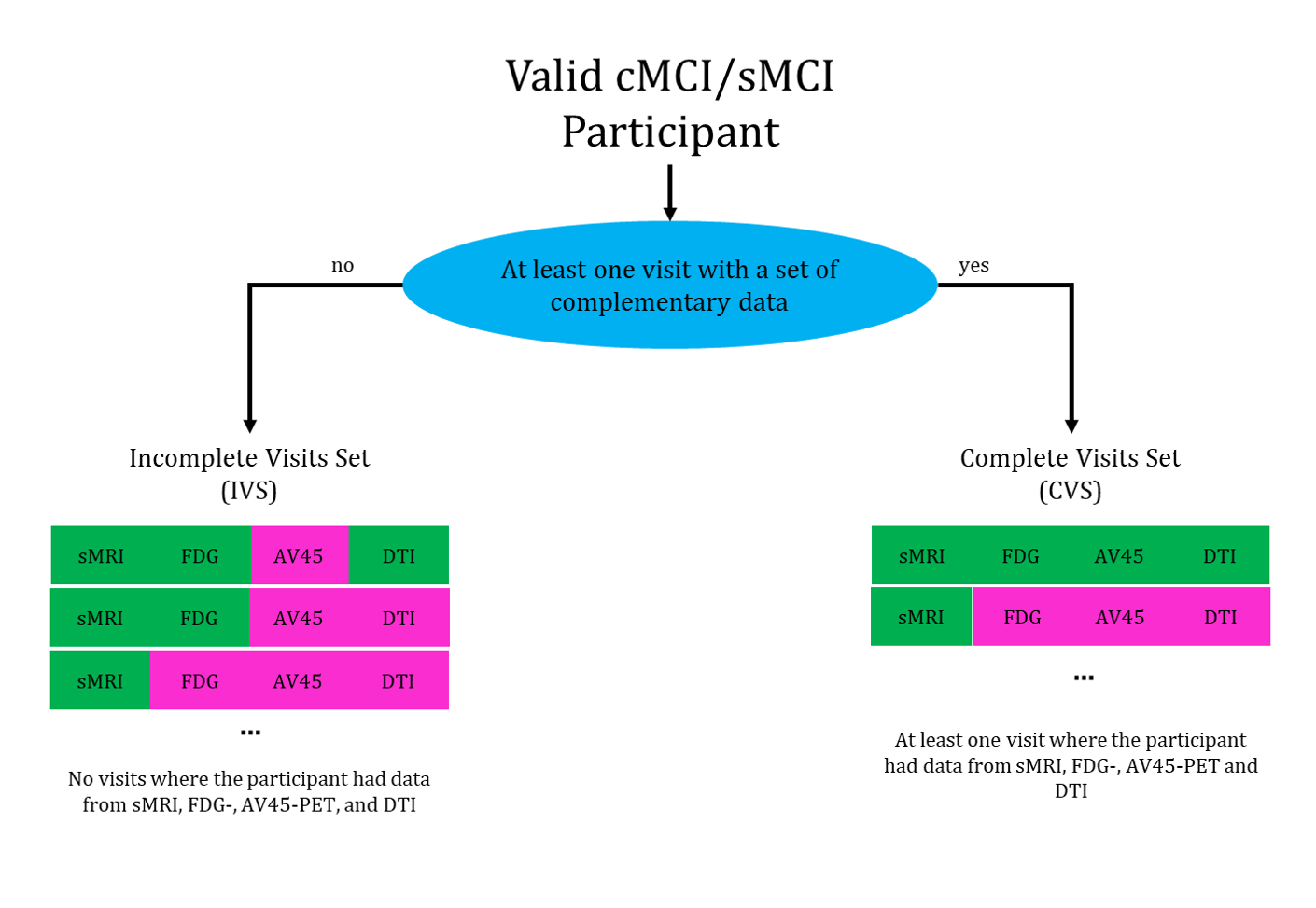


**Supplementary Table 2. Summary of the number of participants considered for each imaging modality as well as the number of scans for each modality in incomplete visits set (IVS) and complete visits set (CVS), depicted by cMCI and sMCI.**

In this table we summarize the total number of scans per imaging modality that we used in this study divided by the two class groups.

|  | **cMCI (n=233)** | **sMCI (n=252)** |
| --- | --- | --- |
|  | **sMRI** | |
| Number of subjects in IVS | 206 | 187 |
| Number of scans in IVS | 939 | 1253 |
| Number of subjects in CVS | 27 | 65 |
| Number of scans in CVS | 122 | 499 |
|  | **AV45** | |
| Number of subjects in IVS | 70 | 123 |
| Number of scans in IVS | 115 | 290 |
| Number of subjects in CVS | 27 | 65 |
| Number of scans in CVS | 47 | 217 |
|  | **FDG** | |
| Number of subjects in IVS | 138 | 142 |
| Number of scans in IVS | 402 | 394 |
| Number of subjects in CVS | 27 | 65 |
| Number of scans in CVS | 35 | 140 |
|  | **DTI** | |
| Number of subjects in IVS | 8 | 30 |
| Number of scans in IVS | 14 | 59 |
| Number of subjects in CVS | 27 | 65 |
| Number of scans in CVS | 106 | 332 |
|  | **All Modalities** | |
| Number of scans in CVS with complementary data | 32 | 95 |

**Supplementary Figure 3. Schematics of the data processing and feature extraction for all imaging modalities.** The imaging modalities considered in the study include structural magnetic resonance imaging (sMRI), fluorodeoxyglucose (FDG) positron emission tomography (PET), florbetapir (AV45-PET), and diffusion tensor imaging (DTI). For each modality, specific feature sets were extracted, including grey matter (GM) and white matter (WM) for sMRI, as well as fractional anisotropy (FA) and mean diffusion (MD) for DTI. These features were utilized to capture relevant information from the different imaging modalities and contribute to the predictive models for Alzheimer's disease conversion from mild cognitive impairment.


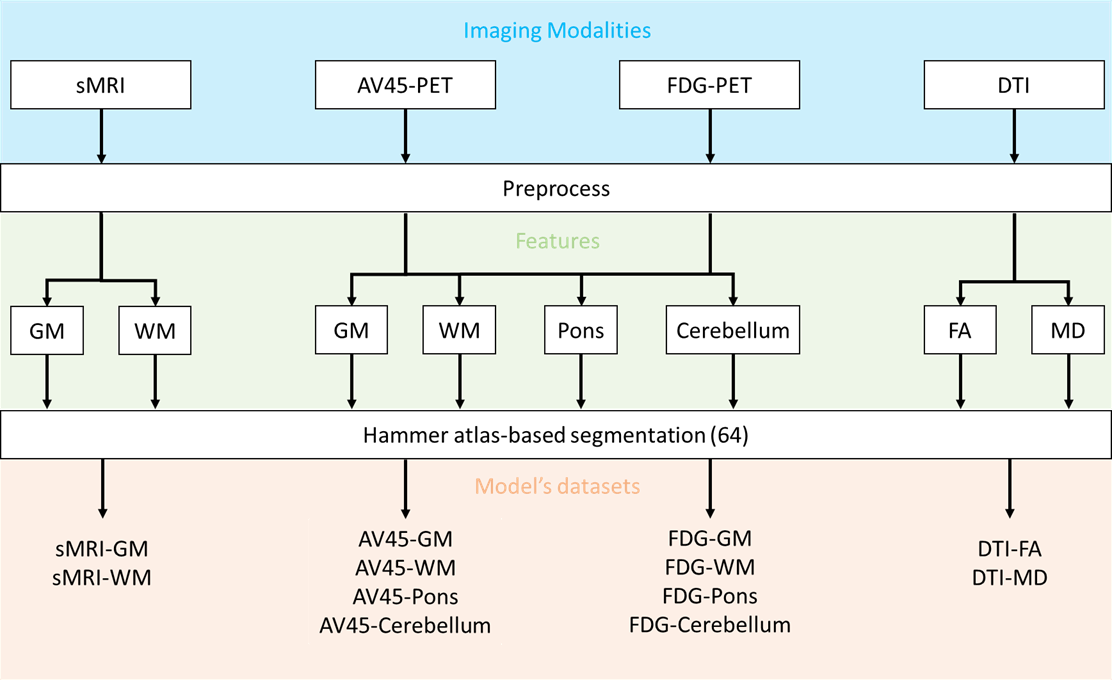


**Supplementary Figure 4. Scheme of the SVM model construction**. Training data was constructed using a random sampling of 30% of the participants present in the Complete Visits Set (CVS) plus all the participants within the Incomplete Visits Set (IVS). The remaining 70% of the participants in the CVS were then used to construct the Test data. Subsequently, a 10-fold cross-validation setup is employed to optimize and extract the optimal c-value using an iterative Bayesian optimization method (optuna). The optimal c-value is then utilized to build a final model using all the train data, which is then validated against the test data. This iterative process ensures the construction of an optimized SVM model with enhanced predictive capabilities for identifying the conversion from mild cognitive impairment (MCI) to Alzheimer's disease. The whole process was repeated 30 times to ensure a fair validation and not introduce bias while performing a favorable split of the CVS participants.


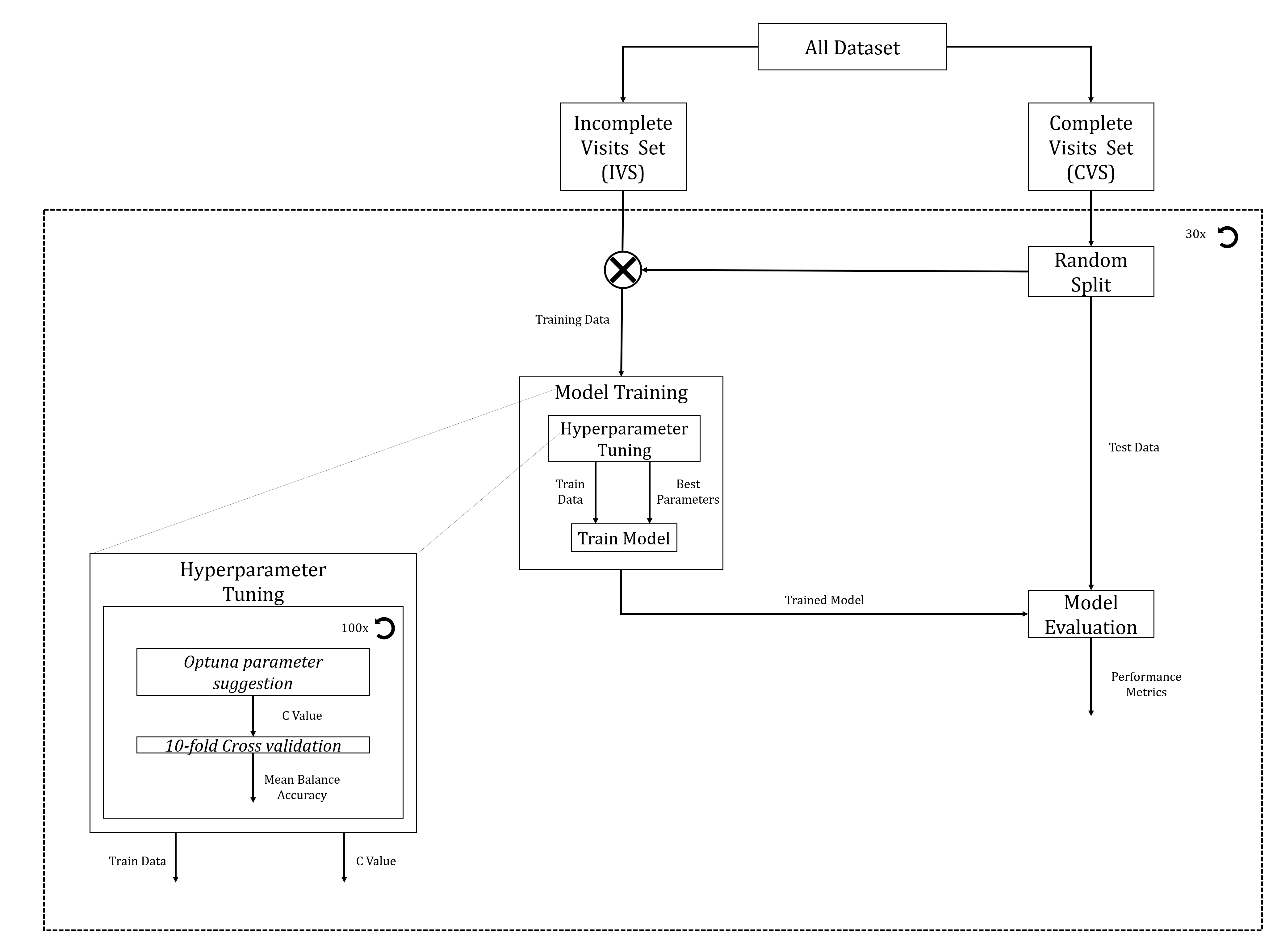


**Supplementary Table 3.** Optimal c-value obtained for each of the models considered in the single-modality approach.

| **Modality** | **Feature** | **Kernel** | **c-value** |
| --- | --- | --- | --- |
| sMRI | GM | Linear | 1.9E-3±2.5E-4 |
|  |  | RBF | 1.3E2±0.3E2 |
|  | WM | Linear | 2.3E-3±4.9E-4 |
|  |  | RBF | 46.83±16.78 |
| AV45 | Cerebellum | Linear | 1.1E-2±3.7E-4 |
|  |  | RBF | 0.74±0.12 |
|  | GM | Linear | 7.7E-3±5.8E-4 |
|  |  | RBF | 0.87±0.09 |
|  | WM | Linear | 6.2E-3±5.9E-4 |
|  |  | RBF | 0.23±0.05 |
|  | Pons | Linear | 1.0E-2±2.6E-3 |
|  |  | RBF | 1.20±0.16 |
| FDG | Cerebellum | Linear | 0.14±0.02 |
|  |  | RBF | 7.09±0.82 |
|  | GM | Linear | 0.10±0.01 |
|  |  | RBF | 3.09±0.42 |
|  | WM | Linear | 0.05±0.01 |
|  |  | RBF | 1.73±0.30 |
|  | Pons | Linear | 1.4E-3±2.8E-5 |
|  |  | RBF | 0.18±0.00 |
| DTI | FA | Linear | 29.52±28.86 |
|  |  | RBF | 1.63±0.67 |
|  | MD | Linear | 1.7E2±0.5E2 |
|  |  | RBF | 98.15±30.63 |

**Supplementary Table 4:** Feature ranking and respective score given by the minimum-redundancy-maximum-relevance (mMRM) feature selection method.

| Rank | sMRI | | | AV45-PET | | | FDG-PET | | DTI | |
| --- | --- | --- | --- | --- | --- | --- | --- | --- | --- | --- |
|  | Region Name | Value | Region Name | | Value | Region Name | | Value | Region Name | Value |
| 1 | Right Amygdala | 1.066667 | Left Putamen | | 1 | Left Posterior Cingular Gyrus | | 1 | Right Corpus Callosum | 6.733333 |
| 2 | Right Posterior Temporal Lobe | 2.2 | Left Accumbens Nucleus | | 3.166667 | Right Posterior Cingular Gyrus | | 4.533333 | Right Postcentral Gyrus | 8.4 |
| 3 | Left Amygdala | 2.866667 | Right Putamen | | 3.4 | Left Hippocampus | | 6.3 | Left Postcentral Gyrus | 11.03333 |
| 4 | Left Hippocampus | 4.1 | Left Inferior Lateral Parietal Lobe | | 4.133333 | Left Inferior Lateral Parietal Lobe | | 7.266667 | Right Superior Parietal Gyrus | 11.16667 |
| 5 | Right Hippocampus | 5.866667 | Left Superior Frontal Gyrus | | 4.433333 | Left Ambient and Parahippocampal Gyri | | 7.4 | Right Precentral Gyrus | 11.46667 |
| 6 | Right Ambient and Parahippocampal Gyri | 6.433333 | Left Superior Parietal Gyrus | | 6.7 | Right Amygdala | | 7.8 | Left Orbito-Frontal Gyri | 12.36667 |
| 7 | Left Posterior Temporal Lobe | 7.833333 | Left Gyrus Rectus | | 6.966667 | Right Hippocampus | | 7.966667 | Right Superior Frontal Gyrus | 12.76667 |
| 8 | Right Fusiform Gyrus | 8.8 | Right Accumbens Nucleus | | 7.666667 | Left Posterior Temporal Lobe | | 8.6 | Left Lingual Gyrus | 12.8 |
| 9 | Right Brainstem | 9.8 | Right Gyrus Rectus | | 10.3 | Left Brainstem | | 8.766667 | Left Posterior Temporal Lobe | 13.83333 |
| 10 | Left Ambient and Parahippocampal Gyri | 10.3 | Right Superior Frontal Gyrus | | 10.53333 | Right Inferior Lateral Parietal Lobe | | 9.633333 | Left Lateral Occipital Lobe | 14.06667 |
| 11 | Right Inferior Middle Temporal Gyri | 12.06667 | Right Middle Frontal Gyrus | | 11.86667 | Left Pallidum | | 10.53333 | Left Insula | 14.16667 |
| 12 | Left Inferior Middle Temporal Gyri | 13.5 | Right Orbito-Frontal Gyri | | 12.9 | Right Ambient and Parahippocampal Gyri | | 11.03333 | Right Lateral Occipital Lobe | 16.13333 |
| 13 | Right Superior Parietal Gyrus | 13.53333 | Right Superior Parietal Gyrus | | 14.26667 | Right Brainstem | | 13.56667 | Right Superior Temporal Gyrus | 16.96667 |
| 14 | Left Inferior Lateral Parietal Lobe | 14.16667 | Left Inferior Frontal Gyrus | | 14.7 | Left Superior Parietal Gyrus | | 14.2 | Right Brainstem | 17.46667 |
| 15 | Right Anterior Medial Temporal Lobe | 14.23333 | Right Inferior Frontal Gyrus | | 15 | Left Middle Frontal Gyrus | | 14.5 | Left Brainstem | 18.36667 |
| 16 | Right Inferior Lateral Parietal Lobe | 15.26667 | Left Superior Temporal Gyrus | | 17.53333 | Right Superior Parietal Gyrus | | 16.8 | Left Precentral Gyrus | 18.73333 |
| 17 | Right Lateral Occipital Lobe | 16.8 | Right Superior Temporal Gyrus | | 17.76667 | Left Inferior Middle Temporal Gyri | | 17.96667 | Right Orbito-Frontal Gyri | 19.66667 |
| 18 | Left Anterior Lateral Temporal Lobe | 18.56667 | Left Orbito-Frontal Gyri | | 18.9 | Right Postcentral Gyrus | | 19.86667 | Left Amygdala | 19.66667 |
| 19 | Right Accumbens Nucleus | 19.06667 | Right Posterior Cinguli Gyrus | | 19.26667 | Right Posterior Temporal Lobe | | 20.9 | Left Accumbens Nucleus | 20.3 |
| 20 | Left Anterior Medial Temporal Lobe | 20.2 | Left Postcentral Gyrus | | 20 | Right Fusiform Gyrus | | 21.3 | Right Posterior Temporal Lobe | 20.5 |
| 21 | Left Superior Parietal Gyrus | 20.56667 | Left Inferior Middle Temporal Gyri | | 20.66667 | Left Amygdala | | 21.53333 | Left Inferior Lateral Parietal Lobe | 21.16667 |
| 22 | Left Superior Temporal Gyrus | 22.63333 | Left Middle Frontal Gyrus | | 20.66667 | Left Thalamus | | 22.5 | Right Amygdala | 21.26667 |
| 23 | Left Lateral Occipital Lobe | 22.66667 | Left Posterior Temporal Lobe | | 21.3 | Left Cerebellum | | 22.53333 | Left Hippocampus | 21.96667 |
| 24 | Left Accumbens Nucleus | 24.13333 | Left Anterior Cingular Gyrus | | 23.46667 | Left Fusiform Gyrus | | 22.86667 | Left Middle Frontal Gyrus | 22.53333 |
| 25 | Left Fusiform Gyrus | 25 | Left Posterior Cingular Gyrus | | 25.06667 | Right Anterior Medial Temporal Lobe | | 23.33333 | Left Superior Parietal Gyrus | 22.56667 |
| 26 | Right Superior Frontal Gyrus | 25.86667 | Right Anterior Cingular Gyrus | | 26.2 | Right Inferior Middle Temporal Gyri | | 23.9 | Right Gyrus Rectus | 23.43333 |
| 27 | Left Brainstem | 27.9 | Right Inferior Lateral Parietal Lobe | | 26.33333 | Right Middle Frontal Gyrus | | 24.06667 | Right Cerebellum | 23.53333 |
| 28 | Left Posterior Cinguli Gyrus | 28.63333 | Right Posterior Temporal Lobe | | 27.13333 | Right Pallidum | | 27.46667 | Left Inferior Frontal Gyrus | 27.2 |
| 29 | Right Anterior Lateral Temporal Lobe | 29.6 | Left Insula | | 27.56667 | Left Anterior Medial Temporal Lobe | | 28 | Right Middle Frontal Gyrus | 27.33333 |
| 30 | Right Insula | 30.56667 | Right Postcentral Gyrus | | 27.7 | Right Lingual Gyrus | | 29.03333 | Right Insula | 27.66667 |
| 31 | Right Superior Temporal Gyrus | 31.53333 | Right Precentral Gyrus | | 31.36667 | Right Cerebellum | | 29.66667 | Left Superior Frontal Gyrus | 27.93333 |
| 32 | Right Lingual Gyrus | 32.96667 | Right Inferior Middle Temporal Gyri | | 33.26667 | Right Gyrus Rectus | | 31.06667 | Right Inferior Lateral Parietal Lobe | 31.43333 |
| 33 | Right Cuneus | 33.6 | Right Anterior Lateral Temporal Lobe | | 33.66667 | Right Accumbens Nucleus | | 32.03333 | Right Inferior Middle Temporal Gyri | 31.8 |
| 34 | Left Gyrus Rectus | 34.03333 | Left Precentral Gyrus | | 33.76667 | Left Postcentral Gyrus | | 32.26667 | Left Cerebellum | 32.03333 |
| 35 | Left Superior Frontal Gyrus | 34.4 | Left Anterior Lateral Temporal Lobe | | 34.5 | Right Precentral Gyrus | | 34.43333 | Right Lingual Gyrus | 32.53333 |
| 36 | Right Thalamus | 34.43333 | Right Insula | | 35.36667 | Left Caudate Nucleus | | 34.56667 | Left Inferior Middle Temporal Gyri | 32.7 |
| 37 | Left Thalamus | 36.36667 | Left Lateral Occipital Lobe | | 35.86667 | Left Precentral Gyrus | | 40.26667 | Left Corpus Callosum | 33.5 |
| 38 | Left Corpus Callosum | 36.46667 | Left Pallidum | | 38.73333 | Left Gyrus Rectus | | 40.33333 | Left Posterior Cingular Gyrus | 34.53333 |
| 39 | Right Postcentral Gyrus | 39.1 | Right Lateral Occipital Lobe | | 39.36667 | Left Superior Frontal Gyrus | | 40.83333 | Left Putamen | 36 |
| 40 | Right Inferior Frontal Gyrus | 40.16667 | Left Fusiform Gyrus | | 39.4 | Right Thalamus | | 41.36667 | Left Anterior Lateral Temporal Lobe | 38 |
| 41 | Right Putamen | 41 | Left Cuneus | | 40.8 | Right Orbito-Frontal Gyri | | 41.4 | Right Anterior Medial Temporal Lobe | 39.53333 |
| 42 | Right Gyrus Rectus | 41.83333 | Left Lingual Gyrus | | 41.86667 | Left Inferior Frontal Gyrus | | 41.4 | Right Fusiform Gyrus | 40.9 |
| 43 | Left Postcentral Gyrus | 42.46667 | Right Cuneus | | 44.66667 | Left Accumbens Nucleus | | 41.93333 | Right Posterior Cingular Gyrus | 41.2 |
| 44 | Left Insula | 42.76667 | Right Pallidum | | 44.8 | Left Lingual Gyrus | | 44.13333 | Right Anterior Lateral Temporal Lobe | 43.23333 |
| 45 | Right Corpus Callosum | 45.6 | Right Fusiform Gyrus | | 45.2 | Left Orbito-Frontal Gyri | | 45.16667 | Right Inferior Frontal Gyrus | 44.16667 |
| 46 | Left Orbito-Frontal Gyri | 46.3 | Right Caudate Nucleus | | 46.93333 | Left Putamen | | 46.83333 | Right Hippocampus | 45.56667 |
| 47 | Left Middle Frontal Gyrus | 47 | Left Caudate Nucleus | | 47.06667 | Right Inferior Frontal Gyrus | | 48.76667 | Left Pallidum | 46.1 |
| 48 | Left Precentral Gyrus | 48.9 | Right Lingual Gyrus | | 47.13333 | Left Lateral Occipital Lobe | | 48.83333 | Left Superior Temporal Gyrus | 48.23333 |
| 49 | Left Inferior Frontal Gyrus | 49.6 | Left Amygdala | | 47.86667 | Left Corpus Callosum | | 49.1 | Right Cuneus | 48.56667 |
| 50 | Right Precentral Gyrus | 50.1 | Right Amygdala | | 49.1 | Right Putamen | | 50.33333 | Left Anterior Cingular Gyrus | 48.66667 |
| 51 | Right Middle Frontal Gyrus | 51.16667 | Right Anterior Medial Temporal Lobe | | 51.23333 | Left Anterior Lateral Temporal Lobe | | 51.16667 | Left Gyrus Rectus | 49.5 |
| 52 | Left Putamen | 51.86667 | Left Anterior Medial Temporal Lobe | | 52.16667 | Right Insula | | 52.43333 | Left Cuneus | 50.23333 |
| 53 | Left Caudate Nucleus | 54.03333 | Left Ambient and Parahippocampal Gyri | | 52.3 | Right Anterior Cingular Gyrus | | 52.83333 | Right Pallidum | 51.23333 |
| 54 | Right Orbito-Frontal Gyri | 54.13333 | Right Thalamus | | 53.56667 | Left Insula | | 53.96667 | Right Thalamus | 51.86667 |
| 55 | Left Lingual Gyrus | 54.56667 | Left Thalamus | | 54.1 | Left Superior Temporal Gyrus | | 54.73333 | Left Thalamus | 52.1 |
| 56 | Right Posterior Cinguli Gyrus | 55.1 | Right Ambient and Parahippocampal Gyri | | 55.6 | Right Superior Frontal Gyrus | | 54.93333 | Right Caudate Nucleus | 52.16667 |
| 57 | Left Cuneus | 56.4 | Left Hippocampus | | 57.8 | Right Cuneus | | 56.03333 | Right Ambient and Parahippocampal Gyri | 55.33333 |
| 58 | Left Pallidum | 56.83333 | Left Cerebellum | | 58.56667 | Right Caudate Nucleus | | 56.4 | Left Ambient and Parahippocampal Gyri | 55.46667 |
| 59 | Right Caudate Nucleus | 57.53333 | Right Brainstem | | 59.7 | Right Corpus Callosum | | 57.33333 | Left Fusiform Gyrus | 57.63333 |
| 60 | Right Pallidum | 57.8 | Right Cerebellum | | 60.2 | Right Lateral Occipital Lobe | | 58.83333 | Left Anterior Medial Temporal Lobe | 57.63333 |
| 61 | Left Anterior Cinguli Gyrus | 59.8 | Right Corpus Callosum | | 60.56667 | Left Anterior Cingular Gyrus | | 59.46667 | Left Caudate Nucleus | 57.86667 |
| 62 | Right Cerebellum | 61.96667 | Right Hippocampus | | 61.76667 | Left Cuneus | | 60.16667 | Right Anterior Cingular Gyrus | 58.06667 |
| 63 | Right Anterior Cinguli Gyrus | 62.36667 | Left Corpus Callosum | | 62.16667 | Right Superior Temporal Gyrus | | 60.23333 | Right Putamen | 58.66667 |
| 64 | Left Cerebellum | 63.56667 | Left Brainstem | | 62.93333 | Right Anterior Lateral Temporal Lobe | | 61.56667 | Right Accumbens Nucleus | 60.33333 |

**Supplementary Table 5. Top 10 ranking features for the different imaging modalities.**

| No. | sMRI | AV45-PET | FDG-PET | DTI |
| --- | --- | --- | --- | --- |
| 1 | Right Amygdala | Left Putamen | Left Posterior Cingulate Gyrus | Right Corpus Callosum |
| 2 | Right Posterior Temporal Lobe | Left Accumbens Nucleus | Right Posterior Cingulate Gyrus | Right Postcentral Gyrus |
| 3 | Left Amygdala | Right Putamen | Left Hippocampus | Left Postcentral Gyrus |
| 4 | Left Hippocampus | Left Inferior Lateral Parietal Lobe | Left Inferior Lateral Parietal Lobe | Right Superior Parietal Gyrus |
| 5 | Right Hippocampus | Left Superior Frontal Gyrus | Left Ambient and Parahippocampal Gyri | Right Precentral Gyrus |
| 6 | Right Ambient and Parahippocampal Gyri | Left Superior Parietal Gyrus | Right Amygdala | Left Orbito-Frontal Gyri |
| 7 | Left Posterior Temporal Lobe | Left Gyrus Rectus | Right Hippocampus | Right Superior Frontal Gyrus |
| 8 | Right Fusiform Gyrus | Right Accumbens Nucleus | Left Posterior Temporal Lobe | Left Lingual Gyrus |
| 9 | Left Ambient and Parahippocampal Gyri | Right Gyrus Rectus | Left Brainstem | Left Posterior Temporal Lobe |
| 10 | Right Brainstem | Right Superior Frontal Gyrus | Right Inferior Lateral Parietal Lobe | Left Lateral Occipital Lobe |
